# Reading Korean historical trauma in China: A qualitative content analysis of Douban reviews of Han Kang’s *Human Acts* and *We Do Not Part*

**DOI:** 10.1371/journal.pone.0361034

**Published:** 2026-11-13

**Authors:** Jiazhao Geng, Delu Kong, Baohu Li

**Affiliations:** 1 School of Asian Studies, Beijing Foreign Studies University, Beijing, China; 2 Faculty of Arts, Shanghai University of Electric Power, Shanghai, China; 3 School of Foreign Languages and Cultures, Nanjing Normal University, Nanjing, China; Universitas Prima Indonesia, INDONESIA

## Abstract

Using 1,704 public Douban reviews, this study examines how Chinese readers respond to Korean historical trauma represented in Han Kang’s *Human Acts* and *We Do Not Part*. We developed a 39-code binary coding frame from a 200-review development subset and froze it before formal corpus-wide coding. Two researchers independently coded a separate 400-review validation set, and a third adjudicated 893 disagreement code-cells to create the Human Reference. DeepSeek-V4-Pro then provided preliminary codes for the remaining 1,304 reviews. One researcher verified all 1,304 reviews, a different researcher independently audited 261 reviews under a prespecified blinded audit, and the third adjudicated 534 audit disagreement code-cells. The final 1,704 × 39 matrix shows that readers most often discuss literary form and aesthetics, ethical and memory-oriented reflection, overall evaluation and reading behavior, and affective or embodied response. Reviews of *We Do Not Part* discuss translation, editions, and reading difficulty more often, whereas reviews of *Human Acts* more often mention sadness, memory and witnessing, state violence, and broader ethical or political concerns. The strongest co-occurrence relationships included translation evaluation with translation-induced reading/comprehension difficulty, memory-oriented ethical reflection with literary witnessing, and language of heaviness or despair with embodied response. These pairwise results show that mediation-related, mnemonic, and affective responses each co-occur with closely related reception patterns within individual reviews. The findings show that Chinese reception of Korean historical-trauma fiction cannot be reduced to overall approval or disapproval. It also varies by work and by the ways readers connect literary form, translation, memory ethics, and affect in their responses.

## 1. Introduction

Historical-trauma fiction can bring readers into contact with violence rooted in histories other than their own. In China, Han Kang’s *Human Acts* (2014) and *We Do Not Part* (2021) offer a clear case for examining that encounter. The novels return, respectively, to the 1980 Gwangju Uprising and the Jeju April 3 incident. Chinese interest in Han Kang predates the 2024 Nobel Prize in Literature, although the award increased her visibility and public discussion [1–3]. Scholarship on these novels has mainly examined how they write historical violence. Park [4], for example, studies affect and postmemory, while Chinese-language criticism has discussed multi-perspectival narration, embodied suffering, state violence, historical writing, memory, and ethical witnessing [5–7]. Chung [8] examines polyphony and postmemory in *Human Acts*, and Gasdia [9] reads both works through gendered memory and intergenerational trauma. Such studies explain much about the representation of Korean historical violence in the novels, but they tell us less about what happens when Chinese readers discuss these narratives in their own words.

This leaves a reception question: how do readers respond to historical trauma rooted in another national context? Research on narrative transportation, absorption, and embodied reading provides useful terms for describing reported reading experience [10–13]. Our material, however, consists of unsolicited online reviews rather than psychometric or experimental measures. We therefore use these concepts with care. References to immersion, narrative transportation, or embodiment describe what reviewers report about reading. They do not show that a validated psychological mechanism occurred.

Chinese scholarship has already traced several aspects of Han Kang’s reception. Hu and Xiong [1] and Y. Wang [2] reconstruct the history and mechanisms of her translation, dissemination, and acceptance, while Zhang [3] examines the interpretation of Han Kang’s works in China within the broader context of Korean literature’s globalization. Most directly relevant to the present study, Zhang and Li [14] analyze Douban comments across Han Kang’s fiction. They find that Chinese readers respond to social and humanistic concerns as well as narrative themes and aesthetic value. Evaluations are mixed, but positive recognition occupies the larger share overall. That work provides a direct platform-based precedent for studying Han Kang’s reception in China. Our comparison is narrower. Instead of treating “Han Kang’s reception in China” as one object, we ask how readers respond to the Korean historical trauma represented in two specific novels and whether the responses differ between them.

Digital social reading platforms offer public, self-selected accounts of reading that lend themselves to systematic analysis [15]. Douban, as one such platform, provides a valuable corpus for this purpose. Its user reviews contain readers’ own descriptions of interpretation, difficulty, evaluation, and emotional response, though they are neither representative of all Chinese readers nor equivalent to experimental observations.

We therefore examine Chinese reception of the Korean historical trauma represented in *Human Acts* and *We Do Not Part* through public Douban reviews. The study uses qualitative content analysis and a coding frame developed from the corpus. It addresses three questions: (RQ1) What reception patterns are observable in Chinese Douban readers’ responses to the Korean historical trauma represented in *Human Acts* and *We Do Not Part*? (RQ2) To what extent do reception patterns differ between the two novels? (RQ3) How do different reception patterns co-occur in Douban reviews?

## 2. Literature review

### 2.1 Research on Han Kang’s historical trauma fiction

Research on Han Kang has expanded rapidly, but much of it still focuses on textual interpretation rather than documented reader response. *The Vegetarian* has prompted extensive discussion of feminism, ecofeminism, corporeality, translation, and world-literary prestige [16,17]. Work on *Human Acts* and *We Do Not Part* has concentrated more on historical violence, postmemory, testimony, and the ethics of remembering. Chung [8], for example, reads *Human Acts* through photography, poetry, intertextuality, and polyphony to ask how a postmemory generation approaches the Gwangju trauma. Park [4] considers both novels as affective and postmemorial engagements with traumatic events not directly lived. These readings point to features that may matter to readers, but they cannot by themselves show how readers in a particular receiving culture respond to them.

Chinese-language criticism has developed a related set of readings. Yang [7] examines the historical writing of violence and suffering in *Human Acts*, with particular attention to multiple perspectives and individual voices within collective violence. H. Wang [6] reads *We Do Not Part* as a recontextualization of Jeju memory through present, personal, and ethical frames, while Dong [5] focuses on state violence, historical writing, and individual resistance. Chung’s [8] study of *Human Acts* also centers on polyphony, intertextuality, and postmemory. Memory, bodily pain, violence, witnessing, and ethical responsibility recur across this scholarship. Reception research lets us ask whether the same concerns appear when readers describe their own encounters with the novels.

A second strand of Chinese scholarship moves from close reading toward circulation and reception. Hu and Xiong [1] survey Han Kang’s translation and acceptance in China. Y. Wang [2] situates her literary world within the history of Chinese translation and dissemination, and Zhang [3] links translation and interpretation to world-literary circulation. Zhang and Li [14] take a further step by analyzing Douban comments. They report broad recognition of Han Kang’s thematic and aesthetic value alongside mixed evaluations. This body of work connects textual interpretation and translation history with platform-based evidence of reader response.

The present study adds to this literature without treating frequency counts as a substitute for close reading. We ask instead whether concerns prominent in Han Kang scholarship, including affect, memory, violence, literary form, translation, and ethical reflection, also appear in reader-generated discourse. The coding also leaves room for responses tied specifically to the act of reading or to platform discussion. Comparing *Human Acts* with *We Do Not Part* matters because a single account of “Han Kang’s reception in China” can hide differences between individual works.

### 2.2 Chinese-language translation, publication, and reception context

The two novels entered Chinese-language circulation under different translation and publication conditions. *Human Acts* circulated in Traditional Chinese as 少年來了, translated by Yin Jiaxuan and published by Azoth Books (漫遊者文化) in Taiwan in 2018 [18]. *We Do Not Part* entered the Simplified Chinese market as 不做告别, translated by Lu Hongjin and published by Jiuzhou Press in 2023 [19]. The editions therefore differ not only in publication date but also in script, market, and publication setting. Recent work on Han Kang’s dissemination in China treats translation and publication as part of the reception context rather than as neutral background [1–3]. At a broader level, Jin and Kim [20] show that the international publication of Korean literature varies across language-specific cultural and market conditions, while Hu [21] reviews methods used in translation-reception research.

Public and scholarly attention to Han Kang’s Chinese-language editions increased after the 2024 Nobel Prize in Literature [1–3]. We do not model media exposure as an explanatory variable. The coding records only what reviewers themselves say about translation, edition, and reading difficulty.

### 2.3 Douban as a digital social-reading platform

Douban is a Chinese user-generated cultural platform for books, film, music, and related activities, launched in 2005. Its book service allows users to record reading, assign ratings, and publish reviews. For this study, the important feature is the availability of persistent, public discussion centered on particular books. Digital social reading is now an established object of literary study [15,22], and Zhang and Li [14] offer a direct precedent for examining Han Kang’s reception through Douban comments. In this article, “Chinese readers” refers operationally to reviewers participating in Chinese-language reception on Douban. We do not infer individual users’ nationality or place of residence.

### 2.4 Empirical approaches to literary reception

Reception theory has long located literary meaning in the encounter between reader and text rather than in the work alone. Jauss’s [23] “horizon of expectations” emphasizes the aesthetic and historical experience that readers bring to a text. Iser’s [24] “implied reader” and Fish’s [25] “interpretive communities” approach interpretation differently, but both place the reader centrally in the production of meaning. Their main limitation for this study is methodological: the reader in classical reception theory is often a theoretical figure rather than an empirically observed person who leaves a public record of response.

Empirical studies of literary reading approach part of this problem through experiments and validated self-report instruments. Narrative transportation, absorption, self-implication, and embodied simulation have all been studied in this way [10–13,26,27]. Hu [21] likewise describes a range of methods for studying translation reception, including surveys, interviews, eye-tracking, and large-scale data analysis. These studies inform our terminology, but the present corpus is different. Douban reviews are unsolicited texts, not validated measures of transportation, absorption, or physiological response. We therefore assign codes such as “immersion” and “embodied response” only when reviewers explicitly describe those experiences. We do not infer a psychological mechanism from wording alone.

Digital social reading provides another kind of evidence. Platforms such as Douban preserve naturally occurring public accounts of reading, including judgments, difficulties, emotions, and comparisons that may not arise in laboratory tasks. What these data offer is naturally occurring reader discourse, not experimental control. Emotional or bodily language, for instance, may reflect felt experience, rhetorical convention, or both. We therefore analyze the reviews as reception discourse and use concepts from reading psychology only as interpretive reference points.

Corporeal and affective language is prominent in the novels and in scholarship on them, which makes the distinction relevant. The analysis asks whether reviewers describe bodily response, immersion, or distance, and whether those descriptions co-occur with other reception patterns. The codes capture public accounts of reading experience. They are not direct measures of physiological embodiment, narrative transportation, or cross-cultural transmission.

### 2.5 Cross-cultural memory as an interpretive framework

Both the representation of trauma in the novels and the reception of historical-trauma narratives across cultural contexts fall within the scope of the analysis. Trauma and cultural-memory scholarship supply terms for interpreting this movement. Caruth [28] describes trauma through belatedness and the difficulty of direct representation, an approach that helps explain fragmentation, repetition, and temporal disruption in trauma narratives. Later scholarship has questioned accounts of trauma that treat it as universally unspeakable or detach it from social context [29,30]. That criticism is relevant here because forms of trauma representation are tied to the cultural, political, and historical conditions under which experiences become speakable.

Cultural-memory theory shifts the emphasis from representation itself to the movement of memory across contexts. Rothberg’s [31] concept of multidirectional memory rejects the idea that historical traumas must compete for public recognition; memories can be placed in relation and take on new meanings. His later account of the “implicated subject” adds an ethical dimension to those encounters [32]. Erll’s [33] “travelling memory” similarly stresses that memories change through translation, appropriation, and recontextualization. These concepts help frame cross-cultural reception, but review-level co-occurrence is not evidence that memory has been transferred.

Explicit cross-cultural, cross-historical, or world-literary positioning is treated as one possible form of transnational reception, not as its definition. Statements that connect Gwangju or Jeju with another history, culture, public-memory frame, or world-literary position can be read alongside work on multidirectional and travelling memory. More general affective, ethical, or mnemonic engagement is not automatically coded as cross-cultural analogy. The corpus cannot reveal a cognitive mechanism of activation, suppression, or transfer, and the analysis does not claim one.

The literature contributes three kinds of support to the analysis. Scholarship on Han Kang identifies recurring questions in her writing of historical trauma. Research on digital social reading shows how public reader discourse can be studied empirically. Work on trauma, memory, and reading psychology provides interpretive concepts, as long as those concepts are not treated as variables directly measured in the reviews. These lines of work are brought together through coding-frame development, human reference coding, verification, audit, and adjudication.

## 3. Materials and methods

### 3.1 Research design

Qualitative content analysis with a fixed coding frame is used [34]. The research questions concern overall reception patterns, differences between the two works, and co-occurrence among reception patterns. They do not specify individual codes, expected prevalences, or expected directions of difference. Candidate codes were developed from a 200-review development subset through inductive, researcher-led reading, with reflexive thematic-analysis principles used only as a text-proximate developmental heuristic. That developmental stage was separate from the final corpus-wide coding. Once the frame had stabilized, we froze a 39-code codebook and recorded the presence or absence of each code at review level. Following qualitative coding methodology, these codes are researcher-defined interpretations of observable discourse rather than naturally occurring or psychologically objective categories [34,35]. Model outputs were auxiliary inputs during codebook development, blind benchmarking, and production pre-coding. Audit-sampling preparation was carried out by researchers. No model output became a final label without human review. S1 File contains the two development-stage codebook prompts (open coding and semantic consolidation), the frozen production prompt and the public-facing operational representation of the frozen 39-code codebook; source-linked illustrative review wording is not redistributed.

The complete review was the unit of analysis, and a review could receive more than one substantive code. C0 (“no codable reception content”) was mutually exclusive with the other 38 codes and was assigned only when no substantive code was present. No evaluative-content screening prompt or screening stage was used in the reported study. All 1,704 reviews in the frozen corpus were retained for formal coding, with C0 representing reviews without substantive reception content. The only development-stage prompts retained in S1 File are the open-coding and semantic-consolidation prompts used for codebook construction; neither was used to exclude reviews. S1 File also contains the frozen production pre-coding prompt and the public-facing operational representation of the frozen 39-code codebook.

### 3.2 Data source, collection, and ethics

The data came from Douban Reading, the Chinese social-reading platform described above. We collected reviews of *Human Acts* and *We Do Not Part* on 20 May 2026. A Python script retrieved the reviews from book-review and comment pages that were publicly accessible at the time. The analysis reported here concerns review text and derived coding variables, not reviewer identity.

The study involved no intervention. Reviewers were not recruited, contacted, or interacted with, and non-public account content was not used in the analysis. The original webpage collection could capture publicly displayed account information, including usernames or profile URLs. These identifiers were not used as analytic variables and were excluded from the final analytic and shared datasets. The manuscript does not reproduce verbatim reviews, and the shared analytical data contain derived coding variables rather than raw review text. These choices reduce the risk of tracing analytical records back to individual platform users.

On September 7, 2026, the College Academic Committee of the School of Asian Studies, Beijing Foreign Studies University reviewed the study’s data source, research design, data-processing procedures, and privacy safeguards and issued a written determination that formal ethics review was not required. The determination was based on the study’s non-interventional analysis of publicly accessible online texts and the absence of recruitment, contact, intervention, surveys, interviews, or experiments.

Public accessibility was treated as the basis for observational access, not as permission to republish platform content without restriction. Raw review text and user-identifying information are not redistributed. When review content is used illustratively, we paraphrase only the meaning relevant to the coding and do not reproduce distinctive reviewer wording.

### 3.3 Corpus construction

The frozen corpus contained 1,704 reviews: 867 for *Human Acts* and 837 for *We Do Not Part*. Reviews with no identifiable reception content received C0 rather than being removed, so the denominator remains fixed across the reported analyses.

### 3.4 Codebook development

Codebook development was inductive and researcher-led. Within the 200-review development subset, models generated open-code and semantic-consolidation proposals as auxiliary inputs. The researchers then revised, split, merged, defined, or discarded those proposals. Exploratory outputs never counted as final labels. Once the coding frame was stable, we froze it and applied the same 39-code codebook deductively across the analytic corpus. This move from inductive development to standardized application follows established practice in qualitative content analysis and qualitative coding [34,35]. The final frame contains C0 and 38 substantive binary codes. For reporting purposes, the 38 substantive codes are arranged into eight predefined code groups (C1-C8). A group is present when at least one member code is present. These groups are descriptive aggregates, not themes, latent constructs, or new inductively generated categories. Each code in the frozen working codebook has a label, operational definition, inclusion and exclusion criteria, boundary rules, and illustrative examples. The public S1 File representation retains the operational fields but omits source-linked example wording to avoid redistributing third-party user text. We did not choose 39 as a target in advance. Codes were retained, combined, or separated according to analytic distinctiveness and the reception questions guiding the study, and infrequent codes were kept when they captured a distinct aspect of reception. The 200 reviews used during codebook development remained in the 1,304-review extension and were recoded under the frozen framework. The separate 400-review validation set did not overlap with the extension and was not used to develop the codebook. The research questions guided the comparisons, but the coding frame was in place before formal corpus-wide coding and was not inferred from the final statistical results. The eight predefined code groups and their member codes are summarized in Table 1.

**Table 1:** Predefined code groups and member codes.

Code group	Group label	Member codes
C1	Affective and embodied responses	C1.1-C1.7
C2	Literary form and aesthetic perception	C2.1-C2.7
C3	Reading and comprehension difficulties	C3.1-C3.4
C4	Knowledge, history, and cognitive gains	C4.1-C4.3
C5	Translation and edition reception	C5.1-C5.3
C6	Ethical, mnemonic, political, and humanistic reflection	C6.1-C6.6
C7	Intertextual, intermedial, and cross-cultural associations	C7.1-C7.3
C8	Overall evaluation and reading behavior	C8.1-C8.5

The eight code groups are predefined summary aggregations of the substantive codes; a group is counted as present when at least one member code is present. They are not treated as themes or latent constructs. C0 is an exclusivity code for reviews with no codable reception content and is not assigned to a substantive code group. The full 39-code operational definitions are provided in S1 File.

### 3.5 Human reference, blind model benchmarking, and full-corpus verification

Three researchers took part in the human coding and adjudication workflow. The 200 reviews used for codebook development (100 from each novel) were first excluded from the validation pool. From the remaining 1,504 reviews, we drew a stratified random sample without replacement of 200 reviews per novel. This produced a separate 400-review validation set with zero overlap with the development subset. Coder A and Coder B independently coded all 400 reviews using the frozen 39-code codebook. Before adjudication, we calculated raw agreement, Cohen’s kappa, positive agreement, and negative agreement, with nonparametric bootstrap 95% confidence intervals from 10,000 paired review-level resamples. Complete code-level results are reported in S1 File (S2 Table). The third researcher adjudicated disagreements and created the locked Human Reference. Coder A and Coder B agreed on 14,707 of 15,600 binary decisions; the third researcher adjudicated the remaining 893 code-cells. The same three-person team then took different roles in the extension stage. One researcher performed full human verification, a different researcher conducted the independent blind audit, and the third adjudicated verifier-auditor disagreements. A researcher who had performed full verification on a review did not independently audit that same review. Human C and Human D in the supplementary audit files are internal labels for the verifier and auditor, not additional researchers.

DeepSeek-V4-Pro was benchmarked against the locked Human Reference in two independent blind runs, without receiving the Human Reference labels. RUN1 produced 397 valid rows and 3 failed outputs (micro F1 = 0.773; macro F1 = 0.763; mean code kappa = 0.739; exact 39-code review match = 31.2%). RUN2 produced 396 valid rows and 4 failed outputs (micro F1 = 0.779; macro F1 = 0.762; mean code kappa = 0.739; exact review match = 32.8%). Failed outputs were excluded from benchmark denominators rather than imputed or recoded as C0. Code-level performance was similar across the two runs under the frozen prompt, but exact-review agreement remained low. The benchmark therefore informed a deliberately restricted role for DeepSeek-V4-Pro: preliminary production pre-coding subject to complete human verification, not final classification. Repeated-run stability and model agreement were not treated as substitutes for human validation. Since model outputs were never final labels, the final labels were grounded in the adjudicated Human Reference, full human verification of the extension, and an independent blind human audit.

DeepSeek-V4-Pro was then applied to the remaining 1,304 reviews under the same frozen framework, with temperature set to 0.0, thinking mode disabled, each review submitted as a separate request, and no conversational history provided. Of the 1,304 attempts, 1,276 yielded valid preliminary code sets and 28 failed the output-validity check. The failed records were kept without DeepSeek labels and were coded directly from the review text during full human verification. One researcher verified all 1,304 extension records, checking and, when needed, modifying the 1,276 valid pre-code sets and coding the 28 failed cases from scratch. A different researcher independently audited 261 reviews (20.0%), selected after production pre-coding under a stratified sampling rule prespecified before production began (random seed 20260829). The sample was stratified by book and by pre-coding any-substantive-code status. For the 1,276 valid DeepSeek outputs, this status was derived from the model pre-code vector. For the 28 DeepSeek failures, a researcher manually assigned the corresponding auxiliary status solely so that the prespecified audit-sampling rule could be applied. The auditor was blinded to model and verifier labels, and the manual sampling field did not change Human C labels or supply final analytic labels. Across 10,179 binary decisions, verifier-auditor cell-level agreement was 94.75% (micro Cohen’s kappa = 0.702). Of the 261 audited reviews, 59 matched exactly across all 39 codes and 202 contained at least one disagreement. The third researcher adjudicated all 534 disagreement code-cells. After adjudication of the substantive codes, C0 was re-derived deterministically from the 38 substantive codes. Nine audited records required a C0 logic correction; all exclusivity checks then passed. The final 1,704 × 39 matrix combined the 400 adjudicated Human Reference reviews with the 1,304 extension reviews, all of which had undergone human verification.

The 400-review Human Reference is a fully human-coded and adjudicated validation subset. Unverified model predictions are not mixed with human labels in the substantive analysis. Every extension review received human verification, a different researcher performed the blind audit, and the third researcher adjudicated every audit disagreement. All prevalence estimates, between-book comparisons, and co-occurrence analyses were calculated from this human-governed final matrix. The 400-review subset functions as a validation and reference set, not as an independent sample for estimating population prevalence (Table 2).

**Table 2:** Human validation, model benchmark, and audit summary.

Validation component	N	Result
Human-reference reliability	Coder A + Coder B	Per-code kappa + bootstrap 95% CI; raw/positive/negative agreement in S1 File
Adjudicated Human Reference	400 reviews	15,600 decisions; 893 disagreement code-cells adjudicated by third researcher
Blind DeepSeek benchmark RUN1	397 valid	Micro F1 0.773; exact-review match 31.2%; mean code kappa 0.739
Blind DeepSeek benchmark RUN2	396 valid	Micro F1 0.779; exact-review match 32.8%; mean code kappa 0.739
Full extension human review	1,304 reviews	1,276 valid pre-codes verified; 28 failed outputs coded manually; all 1,304 human-verified
Independent blind audit	261 reviews	Different researcher (blind auditor); 94.75% cell agreement; micro kappa 0.702; exact match 59/261
Third-researcher audit adjudication	534 code-cells	All verifier-auditor disagreements adjudicated
Final matrix	1,704 reviews	39 binary codes; C0 exclusivity checks passed

### 3.6 Statistical analysis

All statistics were calculated from the locked 1,704 × 39 matrix, with the review as the unit of analysis. Overall and book-specific percentages were calculated for each code and predefined code group, with percentile bootstrap 95% confidence intervals from 10,000 review-level resamples (random seed 20260836). For each of the 39 codes, a 2 × 2 review-level test assessed the association between book and code presence. Fisher’s exact test was used when any expected 2 × 2 cell was below 5, and Pearson’s chi-square test otherwise. The 39 p values were treated as one family and corrected with the Benjamini-Hochberg false-discovery-rate procedure. The same test-selection rule was applied to the eight predefined code groups, and their p values were corrected as a separate family. Between-book differences are reported in percentage points (*We Do* *Not Part* minus *Human Acts*), with bootstrap 95% confidence intervals, odds ratios, and, where applicable, phi or Cramer’s V. Pairwise co-occurrence among the 38 substantive codes is summarized with phi, Jaccard similarity, and lift.

Two sensitivity analyses were also run. First, the 39 code-level between-book comparisons were repeated within the fully human-coded and adjudicated 400-review Human Reference, which contains 200 reviews from each novel, using the same test-selection rule and Benjamini-Hochberg correction. This check asks whether the strongest full-corpus contrasts are also visible in a subset that did not use model-assisted pre-coding. Second, the 167 C0 reviews were removed, and the between-book tests for the 38 substantive codes were repeated (n = 1,537), again with Benjamini-Hochberg correction. Both analyses were secondary checks and did not replace the prespecified full-corpus results.

These tests describe associations in an observational corpus and do not estimate causal effects of either novel. The books differ in publication and translation history, review period, Nobel-related exposure, and other contextual features. The final comparisons also do not adjust for review length, rating, date, or repeated-user clustering. Between-book differences are therefore interpreted here as differences in reception patterns within this Douban corpus, not as effects caused by translation, narrative strategy, historical familiarity, or any other single factor. The de-identified final analytic matrix is provided as S2 Data, and the reproducibility scripts and de-identified auxiliary label matrices are provided in S3 File.

## 4. Results

### 4.1 Overall distribution of final reception codes and predefined code groups

Of the 1,704 reviews, 167 (9.8%; 95% CI 8.4%–11.3%) received C0 because they contained no codable reception content under the frozen rules. At the code-group level, 49.1% of reviews (95% CI 46.7%–51.3%) contained at least one C2 code for literary form or aesthetic perception. The corresponding percentages were 41.4% (39.1%–43.7%) for C6 ethical, mnemonic, political, or humanistic reflection; 38.1% (35.9%–40.4%) for C8 overall evaluation and reading behavior; and 36.7% (34.4%–39.0%) for C1 affective or embodied response. Among individual codes, overall evaluation/value judgment was most frequent (C8.1, 22.4%). Language/style appreciation or perception (C2.1, 17.1%), negative formal/literary evaluation (C2.5, 17.0%), memory/non-forgetting/retelling ethics (C6.2, 15.5%), and narrative/formal appreciation or perception (C2.2, 15.3%) were also common. C0 records the absence of substantive reception content rather than a form of reception, so it is reported here but not plotted in Fig 1, which shows the 38 substantive codes. Complete code-level estimates and bootstrap confidence intervals are reported in S1 File (S9 Table).

**Fig 1:**
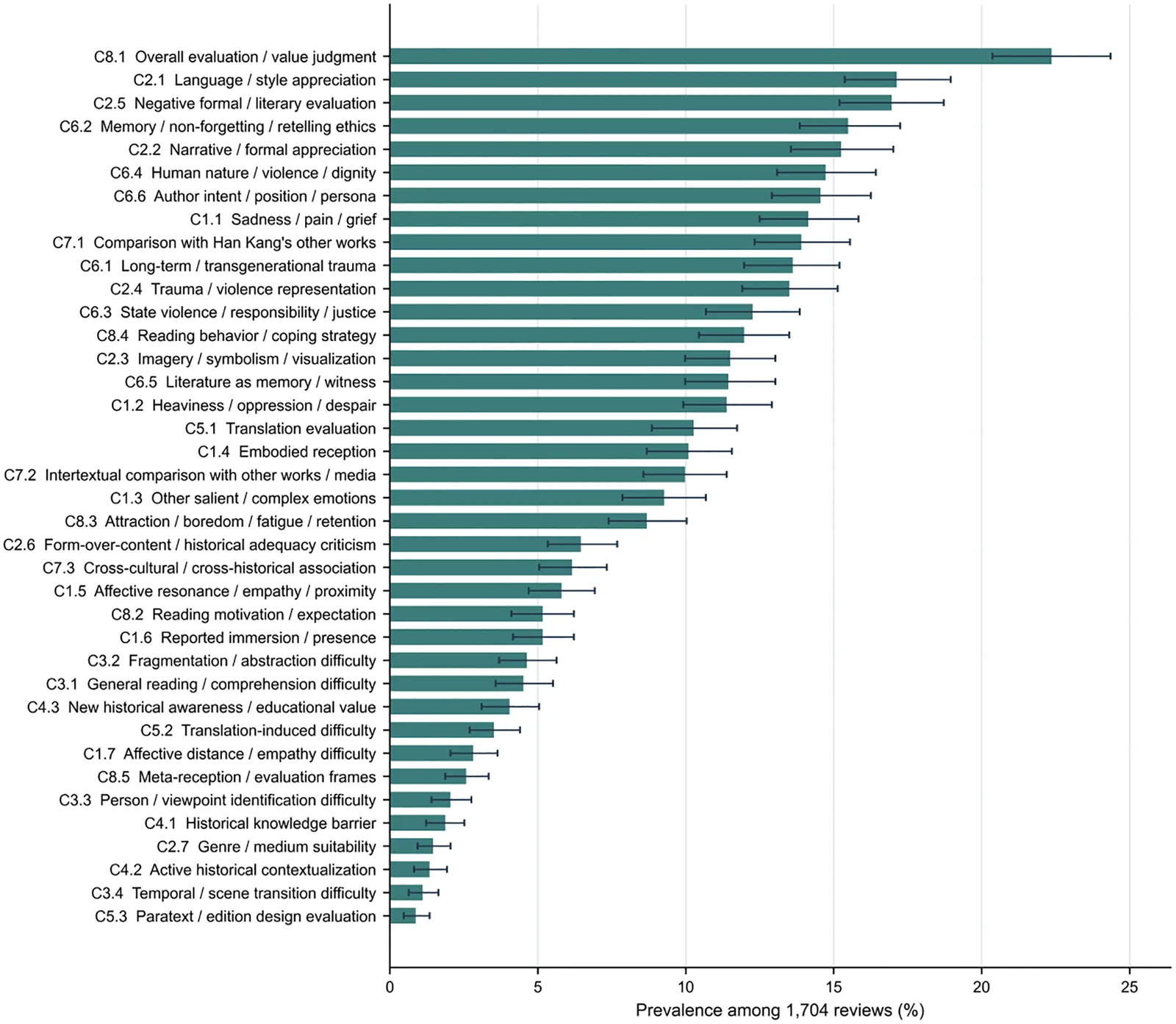
Percentage of reviews assigned each of the 38 substantive reception codes in the final 1,704-review corpus. Horizontal bars show the percentage of reviews assigned each code; whiskers show percentile bootstrap 95% confidence intervals. C0 (no codable reception content; 9.8%) is reported in the text and in S1 File (S9 Table), but is not plotted because it is an exclusivity code rather than a substantive reception category. English code labels in the figure are abbreviated for display; complete code names and operational definitions are provided in S1 File.

### 4.2 Differences between novels across predefined code groups

At the predefined code-group level, the two novels show different reception patterns (Table 3, Fig 2). Reviews of *We Do Not Part* more often contain at least one C2 literary-form/aesthetic code (+5.7 percentage points, q = 0.024), C3 reading/comprehension-difficulty code (+15.1 points, q < 0.001), C4 knowledge/history/cognitive-gain code (+2.9 points, q = 0.019), and C5 translation/edition code (+16.2 points, q < 0.001). Reviews of *Human Acts* more often contain at least one C1 affective/embodied code (10.8 points higher; q < 0.001) and C6 ethical/mnemonic/political/humanistic-reflection code (22.0 points higher; q < 0.001). C7 and C8 do not differ after FDR correction. These are differences observed in this corpus; the analysis does not identify which textual, translational, temporal, or readership factors produced them.

**Table 3:** Percentages of reviews assigned to each predefined code group and between-book comparisons.

Code group	Group label	*We Do Not Part*	*Human Acts*	Difference (pp)	q
C1	Affective and embodied responses	31.2%	42.0%	−10.8	<0.001
C2	Literary form and aesthetic perception	52.0%	46.3%	+5.7	0.024
C3	Reading and comprehension difficulties	18.6%	3.6%	+15.1	<0.001
C4	Knowledge, history, and cognitive gains	7.5%	4.6%	+2.9	0.019
C5	Translation and edition reception	19.4%	3.1%	+16.2	<0.001
C6	Ethical, mnemonic, political, and humanistic reflection	30.2%	52.2%	−22.0	<0.001
C7	Intertextual, intermedial, and cross-cultural associations	24.5%	25.6%	−1.1	0.681
C8	Overall evaluation and reading behavior	38.4%	37.8%	+0.5	0.825

Difference = *We Do Not Part* minus *Human Acts*. q values are Benjamini-Hochberg FDR-adjusted across the eight code-group comparisons. Full p-values, bootstrap CIs, odds ratios, and effect sizes are in S1 File (S10 Table).

**Fig 2:**
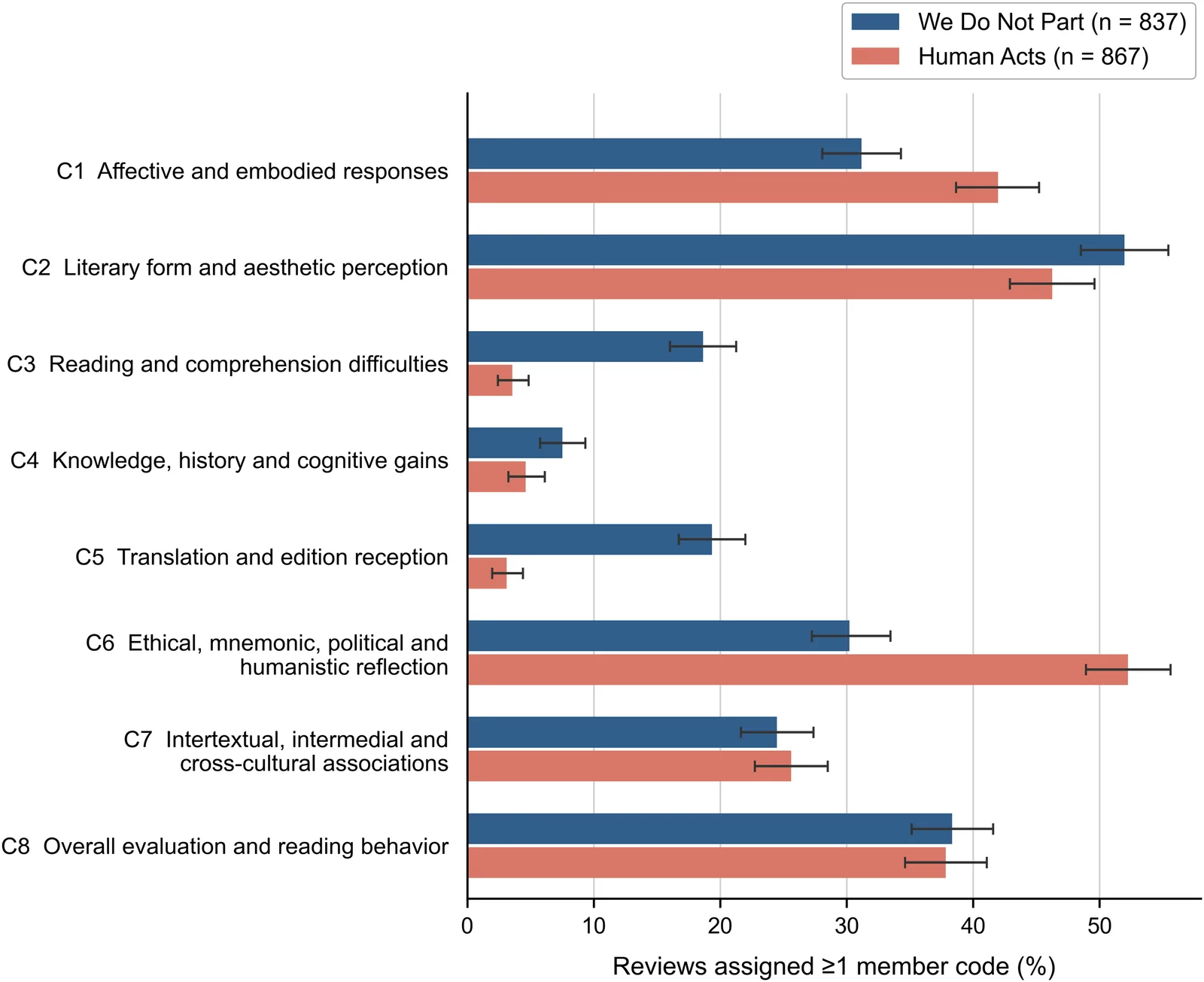
Percentages of reviews coded in each of the eight predefined code groups by novel. A group is counted as present when at least one member code is present. Blue bars indicate *We Do Not Part* and red bars indicate *Human Acts*; whiskers show percentile bootstrap 95% confidence intervals. Between-book q values use Benjamini-Hochberg false-discovery-rate correction across the eight code groups.

### 4.3 Validation and audit outcomes

Table 2 and Fig 3 show why model outputs were used as preliminary rather than final labels. DeepSeek-V4-Pro produced similar code-level results in the two blind runs, but exact 39-code review matches were 31.2% and 32.8%. In production pre-coding, 1,276 of 1,304 attempts returned valid preliminary code sets. The 28 failed outputs were kept without DeepSeek labels and coded directly during full human verification. One researcher verified all 1,304 extension records. A different researcher then independently audited 261 reviews while blinded to model and verifier labels; 202 of those reviews had at least one verifier-auditor disagreement. The third researcher adjudicated all 534 discordant code-cells. We then re-derived C0 deterministically, which required correction in nine audited records. The final extension labels thus come from full human verification, with adjudicated decisions replacing disputed audit labels.

**Fig 3:**
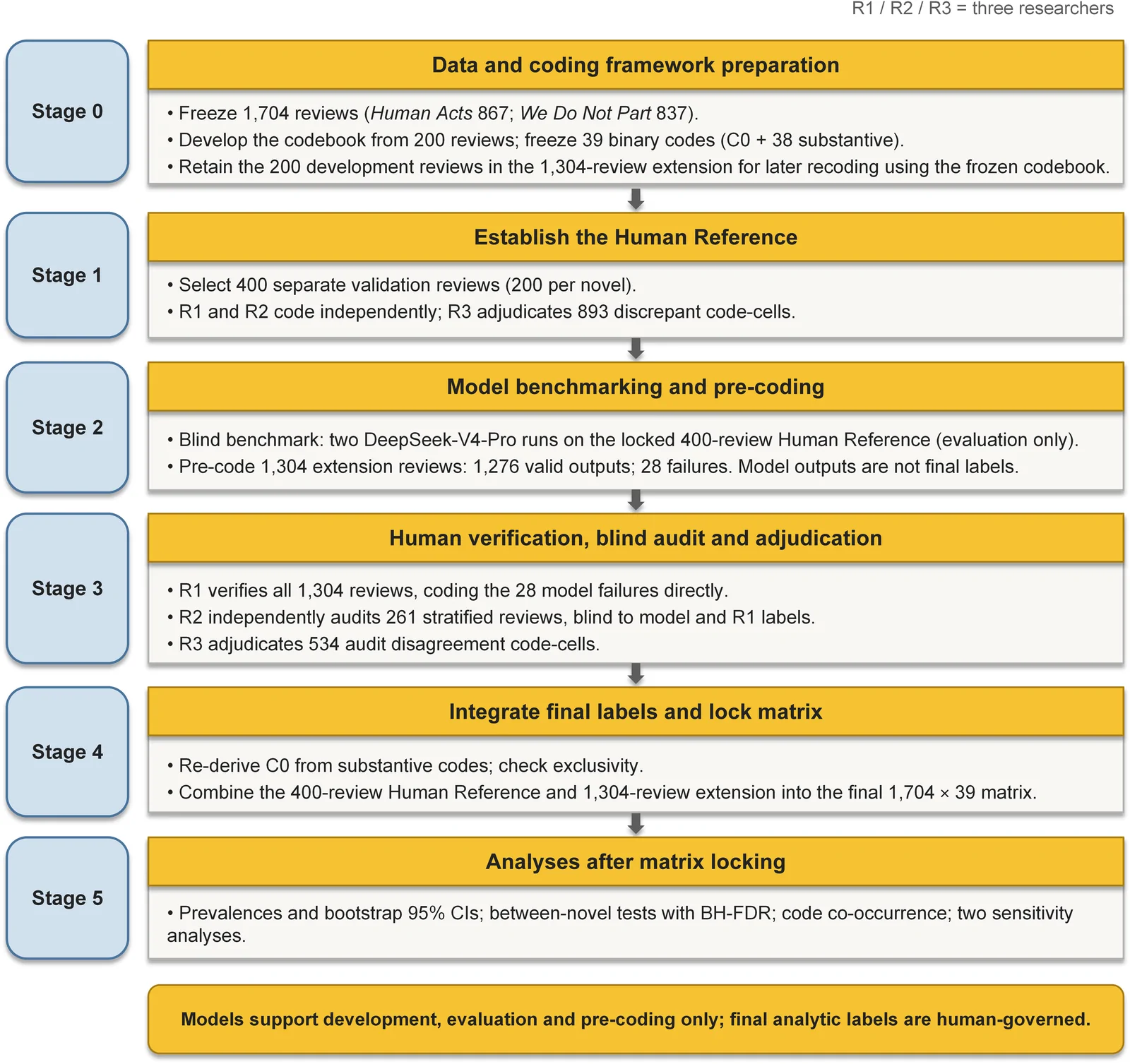
Coding, verification, audit, and adjudication workflow. Three researchers (R1/R2/R3) occupied different roles across stages. Two researchers independently coded the 400-review Human Reference and a third adjudicated their disagreements. For the 1,304-review extension, DeepSeek-V4-Pro was run for preliminary pre-coding (1,276 valid outputs; 28 failed outputs); one researcher verified all 1,304 records and coded failed cases directly, a different researcher independently audited 261 reviews while blinded to model and verifier labels, and the third adjudicated audit disagreements before the final 1,704 × 39 matrix was constructed. The blind-benchmark branch in the figure is evaluation-only: it measures agreement between DeepSeek and the Human Reference and neither generates nor transmits final analytic labels.

### 4.4 Code-level between-book differences

Code-level comparisons show that the group differences were unevenly distributed across their member codes (Fig 4; S1 File (S9 Table)). For *We Do Not Part*, the largest positive differences were translation evaluation (C5.1, + 15.7 percentage points), comparison with Han Kang’s other works (C7.1, + 8.4), fragmentation/abstraction/jumpiness difficulty (C3.2, + 7.8), reading attraction, boredom, fatigue, and retention (C8.3, + 7.6), general reading/comprehension difficulty (C3.1, + 7.1), and translation-induced difficulty (C5.2, + 6.7). *Human Acts* had higher percentages for human-nature/violence/dignity/existential reflection (C6.4, 16.3 percentage points higher), state-violence/responsibility/justice reflection (C6.3, 13.8 higher), sadness/pain/grief (C1.1, 9.5 higher), appraisal of trauma/violence representation (C2.4, 8.2 higher), and literature as memory/witness/historical writing (C6.5, 8.2 higher). Cross-cultural/cross-historical association and world-literary positioning (C7.3) is also relevant to the transnational focus of the study. It appeared in 105 reviews overall (6.2%), including 3.7% of *We Do Not Part* reviews and 8.5% of *Human Acts* reviews (difference = −4.8 percentage points, q < 0.001). The historical knowledge barrier code (C4.1) did not remain significant after FDR correction (q = 0.086). We therefore do not attribute the broader C3/C5 differences to historical unfamiliarity alone.

**Fig 4:**
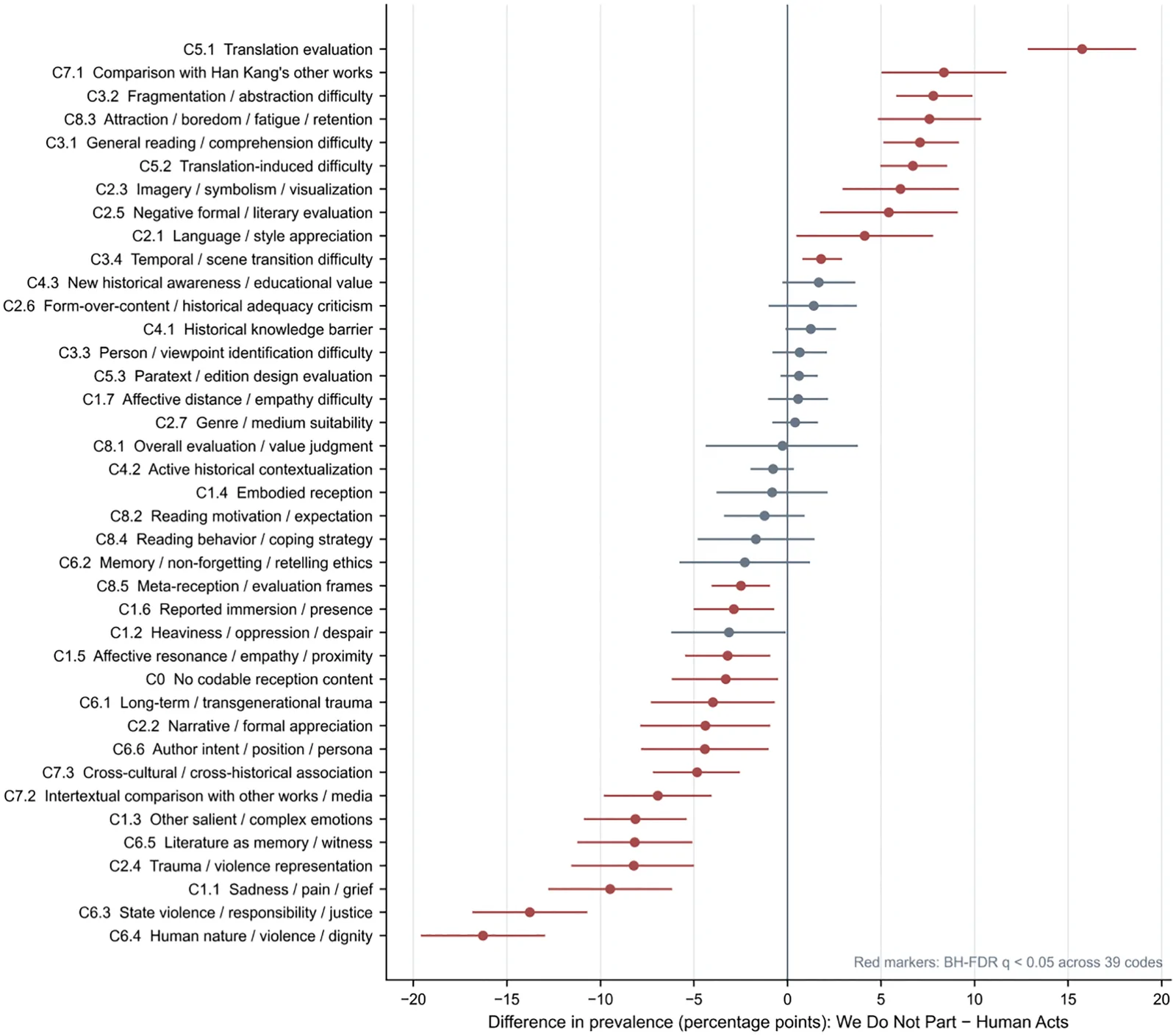
Code-level differences between the two novels. Difference = *We Do Not Part* minus *Human Acts* in the percentage of reviews assigned each code; positive values indicate a higher percentage in *We Do Not Part* and negative values a higher percentage in *Human Acts*. Whiskers show percentile bootstrap 95% confidence intervals. Statistical significance uses Benjamini-Hochberg false-discovery-rate correction across the 39 code comparisons; complete values are in S1 File (S9 Table). Red markers indicate FDR-adjusted q < 0.05 and gray markers indicate results that do not meet that threshold. English code labels are abbreviated for display; complete code names are provided in S1 File.

The sensitivity analyses directionally corroborated several of the largest between-book contrasts, although fewer code-level differences remained significant in the smaller fully human-coded Human Reference subset. Within the fully human-coded 400-review Human Reference, nine codes remained significant after correction. The strongest contrasts included translation evaluation (C5.1, + 17.5 percentage points), human-nature/violence/dignity reflection (C6.4, −19.5), state-violence/responsibility/justice reflection (C6.3, −17.0), and general reading/comprehension difficulty (C3.1, + 11.0), all in the same direction as the full-corpus estimates. In the second sensitivity analysis, removing C0 and repeating the 38 substantive-code tests retained 23 of the 24 substantive codes that were significant in the primary analysis. C2.1 moved above the false-discovery-rate threshold (q = 0.080), while C1.2 moved below it (q = 0.033); effect directions did not change. Thus, several of the largest contrasts were not unique to the model-assisted extension or to inclusion of C0 reviews, while marginal findings remained sensitive to sample size and the significance threshold.

### 4.5 Co-occurrence of reception patterns

When the 38 substantive codes were ranked by absolute phi, several of the strongest pairwise relationships were positive and joined codes that were semantically related but operationally distinct. The strongest was translation evaluation with translation-induced reading/comprehension difficulty (C5.1 + C5.2, phi = 0.523). Other prominent pairs were memory/non-forgetting/retelling ethics with literature as memory/witness/historical writing (C6.2 + C6.5, phi = 0.468), understanding long-term/transgenerational trauma with memory/non-forgetting/retelling ethics (C6.1 + C6.2, phi = 0.369), negative formal/literary evaluation with form-over-content/historical-adequacy criticism (C2.5 + C2.6, phi = 0.359), and heaviness/oppression/despair with embodied reception (C1.2 + C1.4, phi = 0.346). These pairwise results show that mediation-related, mnemonic, and affective responses each co-occur with closely related reception patterns within individual reviews. The full 38 × 38 phi matrix is shown in S1 File (S2 Fig). These are descriptive relationships. They may partly reflect overlap in code content, review length, code frequency, coding thresholds, or shared rhetorical conventions.

## 5. Discussion

### 5.1 Multidimensional reader reception

The 39-code analysis shows that Chinese Douban readers respond to these two historical-trauma novels on several fronts. Literary form and aesthetics are common concerns, but readers also discuss memory, ethics, politics, affect, value, and the experience of reading. Zhang and Li [14] similarly report that Douban readers respond to both Han Kang’s thematic and social concerns and her aesthetic value, with positive recognition prominent overall. The present analysis complicates that summary. Positive judgment can appear alongside difficulty, translation criticism, emotional burden, or ethical reflection, both across the corpus and within a single review. Reception theory offers a useful way to read this heterogeneity: literary meaning emerges through historically situated relations between readers and texts and through interpretive communities, rather than as a fixed effect of the text itself [23,25]. A label such as “positive reception” therefore captures only part of what readers are doing. For transnational reception research, it is more informative to describe the response patterns that coexist than to reduce reception to a single evaluative direction.

Concepts from the psychology of reading are useful for describing some of these responses, but the evidence in the reviews sets clear limits. C1.4 records embodied language, and C1.6 records explicit reports of immersion or presence. Neither code measures physiological embodiment or validates narrative transportation. Keeping that distinction allows comparison with experimental research on reading without treating reviewers’ wording as a psychological measure.

C7.3 provides a more specific view of the study’s transnational dimension. Cross-cultural/cross-historical association and world-literary positioning occurred in 105 reviews, or 6.2% of the corpus, and appeared more often in *Human Acts* than in *We Do Not Part*. Explicit positioning across national, historical, cultural, or world-literary frames was therefore present but not dominant. The absence of C7.3 should not be interpreted as evidence that a review lacked any transnational dimension, because C7.3 operationalizes only explicit cross-cultural, cross-historical, or world-literary positioning. The present coding cannot determine whether less explicit forms of transnational engagement occurred. At the level of reader discourse, this pattern can be read alongside Erll’s account of memory moving through recontextualization and Rothberg’s account of histories entering relational fields of remembrance [31,33]. The reviews document such recontextualization as discourse; they do not demonstrate cognitive transfer of memory.

### 5.2 Reception differences between the two novels

The clearest differences between the two novels concern translation, reading difficulty, and ethical or memory-oriented response. Reviews of *We Do Not Part* discuss translation or edition and reading or comprehension difficulty more often. Reviews of *Human Acts* contain more ethical, political, memory-oriented, and affective reflection. Existing scholarship helps place these differences in context. Studies of Han Kang’s translation and dissemination in China show that editions and publication history are part of her reception there [1–3]. Jin and Kim [20] likewise show that the international publication of Korean literature varies across language-specific cultural and market conditions, and Hu [21] describes translation reception as an interaction among texts, readers, and receiving contexts. In our corpus, translation is especially visible in reviews of *We Do Not Part* because readers often evaluate it and sometimes connect it with difficulty. That pattern does not establish that translation quality caused the difficulty. It shows that translation and edition became explicit objects of reception. Reviews of *Human Acts*, in contrast, more often refer to sadness, memory and witnessing, state violence, and human dignity. Critical readings that focus on polyphonic testimony, postmemory, inherited trauma, and the ethical implications of historical violence [4,8,9] align with this pattern. The overlap matters because the same concerns appear in criticism and in ordinary reader reviews, not because scholarly interpretation determines popular response.

Reviews of *We Do Not Part* also resist a single explanation for difficulty. H. Wang [6] reads the novel through the recontextualization of Jeju memory in present, personal, and ethical frames, while Dong [5] discusses historical writing, state violence, and individual resistance. Douban reviewers engage with these issues but also comment frequently on textual opacity, fragmentation, translation, and edition-related matters. The specific historical-knowledge barrier code (C4.1) is not significant after FDR correction, so this result does not provide sufficient evidence to attribute the broader C3/C5 difference to historical unfamiliarity alone. “Difficulty” is also not a single construct in the coding. Readers distinguish general comprehension problems, fragmentation or abstraction, and translation-related difficulty, and these do not show identical between-book patterns. The translation-related codes record readers’ own evaluations of translations and editions, not independent expert judgments of translation quality. The contrast between the two works therefore supports keeping translation/edition mediation separate from historical and ethical engagement rather than combining them in one measure of “difficulty” or “acceptance.”

### 5.3 Co-occurring reception patterns

The co-occurrence results describe relationships among coded reception patterns rather than opposed modes of reading. Several pairings are particularly clear. Translation-induced reading/comprehension difficulty was closely associated with translation evaluation: 56 of the 60 reviews coded for translation-induced difficulty also carried translation evaluation. Memory/non-forgetting/retelling ethics co-occurred with language about literature as memory, witness, or historical writing. Understanding of long-term or transgenerational trauma also co-occurred with memory ethics. In some reviews, then, historical understanding appears alongside questions of remembering, retelling, and preserving testimony. Work on postmemory and literary witnessing in Han Kang [4,8] and cultural-memory accounts in which remembrance is mediated and recontextualized rather than passed on intact [31,33] offer a relevant frame. Heaviness, oppression, or despair also co-occurred with embodied response, so affective burden is sometimes expressed in bodily terms. Research on absorption and embodied reading provides a useful comparison [11,13], but these reviews cannot establish physiological simulation. Some reviewers, for example, criticized the translation while attributing disrupted comprehension to syntax, phrasing, or lexical choices in the Chinese text. Others connected the responsibility to remember and retell historical suffering with literature’s role in preserving testimony. Still others described emotional heaviness through bodily terms such as breathlessness, cold, or physical discomfort.

These pairwise results show how different reception patterns can occur within the same review. Translation-related difficulty can accompany translation evaluation, memory can appear with witnessing, and affect can be expressed through bodily experience. The analysis does not treat these combinations as stable reader types or as evidence of an unobserved psychological mechanism. Phi gives no causal direction and cannot show cognitive competition. Co-occurrence may also vary with review length, marginal code prevalence, coding thresholds, and rhetorical convention. Its value here is narrower: it shows how analytically distinct responses are combined within reviews, while leaving the mechanisms behind those combinations unresolved.

### 5.4 Human verification of model-assisted coding

The main methodological issue is how model assistance and human judgment were divided. The corpus-wide analysis uses qualitative content analysis with a frozen coding frame; principles of reflexive thematic analysis informed only the earlier inductive development of candidate codes [36,37]. Formal coding used the fixed frame, independent human reference coding, reliability assessment, full human verification, an independent audit, adjudication, and descriptive and inferential comparisons. That structure is consistent with the use of coding frames in qualitative content analysis [34]. Models generated development-stage proposals and production pre-codes and were evaluated in blind benchmarks, but human researchers defined the coding frame and reference standard, verified every extension record, carried out the independent audit, and adjudicated disagreements. Saldaña [35] similarly treats generative AI as potentially useful for descriptive or first-pass coding while keeping interpretation, contextual judgment, and analytic responsibility with researchers. The validation results support treating model output as a fallible auxiliary input rather than an analytic decision. The benchmark results motivated a deliberately restricted role: preliminary coding subject to complete human verification, especially because exact-review matches were much lower than code-level agreement. In production, 28 DeepSeek attempts did not return valid code sets, so the human verifier coded those reviews directly. For those 28 rows, a researcher later assigned the auxiliary audit-sampling status manually; this sampling-only field did not supply final labels. The independent audit found at least one verifier-auditor disagreement in 202 of 261 audited reviews. Model assistance can therefore reduce the burden of preliminary coding, but final analytic decisions still require auditable human judgment.

Development-stage open-coding and semantic-consolidation proposals from the 200-review development subset remained separate from final corpus-wide coding. They did not supply final labels or determine inclusion in the final analytic corpus. All 1,704 reviews remained in the analysis, with C0 used for reviews that contained no substantive reception content. DeepSeek-V4-Pro was the production pre-coding model for the 1,304-review extension. It returned valid pre-code sets for 1,276 attempts; the 28 failed attempts remained without DeepSeek labels and were coded manually during full human verification. As described in Methods, the manually assigned auxiliary sampling-status field for those failed rows did not change human labels or enter the final analytic matrix. Keeping development-stage proposals separate from production coding prevents exploratory model outputs from becoming hidden differences in the final analytic variables.

### 5.5 Limitations and directions for future research

Several limitations should be kept in view. Douban is one platform, and its public reviewers are a self-selected group rather than a representative sample of all Chinese readers. The between-book comparisons are observational and are not adjusted for review length, rating, date, repeated users, publication history, translation edition, or Nobel-related exposure. Significant differences therefore describe this corpus rather than causal effects of the novels. Some low-frequency codes have lower kappa despite high raw agreement, a known issue with chance-corrected reliability measures. S1 File accordingly reports code-level kappa confidence intervals together with positive and negative agreement rather than presenting reliability as uniformly high. Findings involving codes with lower pre-adjudication reliability should therefore be interpreted more cautiously than findings based on more reliably distinguished codes. Every extension record received human verification, but only the prespecified 261-review audit subset received a second independent blind human coding before adjudication. Model behavior may also change across versions and providers, so exact API replication can remain time-sensitive even when the frozen prompt, parameters, model role, and audit chain are documented. Finally, review language cannot establish narrative transportation, physiological embodiment, empathy, or other psychological mechanisms. The source material is public third-party user content. To limit privacy and redistribution risks, the manuscript does not reproduce verbatim reviews or direct identifiers, and the shared dataset contains only derived analytic variables.

## 6. Conclusion

Based on a human-governed 1,704 × 39 review-by-code matrix, this study examines how Chinese readers on Douban respond to the Korean historical trauma represented in Han Kang’s Human Acts and We Do Not Part. Earlier Chinese scholarship has documented Han Kang’s translation, dissemination, and broad reception. By focusing on two historical-trauma novels, the present analysis shows more specifically what reviewers discuss. Literary form, historical memory, ethical and political reflection, affect, evaluation, and reading behavior all appear prominently. The two novels also differ. Reviews of We Do Not Part contain more discussion of translation or edition and reading difficulty, whereas reviews of Human Acts refer more often to sadness, memory and witnessing, state violence, and broader ethical-political concerns. Cross-cultural/cross-historical association and world-literary positioning occur in the corpus but are relatively uncommon overall, so transnational reception should not be equated with explicit analogy to one’s own history. The co-occurrence results add another part of the picture: translation-induced reading/comprehension difficulty can appear with translation evaluation, memory-oriented ethical reflection with literary witnessing, and language of heaviness or despair with embodied response. Taken together, these pairwise associations show that mediation-related, mnemonic, and affective patterns each connect with other reception features within individual reviews. These findings support an account of cross-cultural literary reception that attends to differences between works and to the literary, translational, mnemonic, ethical, and affective concerns that readers combine, rather than reducing reception to a single label of acceptance. Models remained auxiliary throughout the method. They supported candidate-code development, benchmarking, and production pre-coding, while audit-sampling preparation, the coding frame, Human Reference, full human verification, independent blind audit, and adjudication remained under human control.

## Supporting information

S1 File Supplementary material. This file provides the development-stage and production prompt records, the public-facing operational representation of the frozen 39-code codebook, Human Reference construction and reliability/adjudication evidence, blind DeepSeek-V4-Pro benchmarks, the independent extension audit, final-matrix provenance, sensitivity analyses, complete statistical results, Supplementary Fig S2, and documentation of the reproducibility materials.
(DOCX)

S2 Data English-language analytic matrix. This de-identified dataset contains 1,704 review-level records, English book titles, the 39 final binary code variables, final_label_provenance, and independent_audit_applied. It contains no verbatim review text or direct user identifiers.
(CSV)

S3 File Reproducibility code archive. This archive contains README.md, requirements.txt, modular Python scripts, and de-identified auxiliary coding matrices used to reproduce the human-reliability, model-benchmark, audit, quality-control, sensitivity, co-occurrence, and final statistical analyses. It contains no verbatim Douban review text, direct user identifiers, API credentials, or network-calling code.
(ZIP)
