# Supplementary material for: Reading Korean historical trauma in China: A qualitative content analysis of Douban reviews of Han Kang’s Human Acts and We Do Not Part
Source: PLoS One. 2026 Nov 13;21(11):e0361034. doi: 10.1371/journal.pone.0361034 (PMC13766255; doi:10.1371/journal.pone.0361034)
Supplement: Supplementary file 1 [file pone.0361034.s001.docx]

***Supplementary Material***

Reading Korean historical trauma in China: A qualitative content analysis of Douban reviews of Han Kang’s *Human Acts* and *We Do Not Part*

This document provides the final model-assisted coding protocol, two development-stage codebook prompts retained for reproducibility, the public-facing operational representation of the frozen 39-code codebook, construction of the 400-review Human Reference and its human reliability/adjudication evidence, two blind DeepSeek-V4-Pro benchmarks, the independent extension audit, final-matrix provenance, sensitivity analyses, complete final statistical results for the 1,704-review corpus, Supplementary Fig S2, and documentation of the accompanying reproducibility code. Source-linked illustrative review wording embedded in historical prompt/codebook materials is not redistributed in this public supplement; code names, definitions, inclusion/exclusion criteria, boundary rules, and all reported analytic results are retained. Final analytic labels are human-governed: model outputs were auxiliary inputs for codebook development, benchmarking, and pre-coding and were not treated as final analytic decisions. Audit-sampling preparation was carried out by researchers.

# 1. Large language models and final analytic configuration

Large language models were used only to generate codebook-development proposals, produce outputs evaluated in the blind benchmarks, and provide production pre-codes. They did not make final analytic decisions. The final 39-code system was frozen before formal corpus-wide coding. The 400-review Human Reference set was independently coded by two researchers and adjudicated by a third. The remaining 1,304 reviews received DeepSeek-V4-Pro pre-coding where valid, followed by 100% human verification, a prespecified 261-review independent blind human audit, and third-researcher adjudication of audit disagreements. The 28 failed DeepSeek outputs were coded directly from review text during full human verification.

**Supplementary Table S1. Model and decision sources in the final analytic pipeline**

| **Stage** | **Task** | **Model / decision source** | **Temperature** | **Role** |
| --- | --- | --- | --- | --- |
| Codebook development | Open/inductive coding | DeepSeek-V4-flash | 0.3 | Proposal only; researcher-reviewed |
| Codebook development | Semantic clustering / consolidation proposals | DeepSeek-V4-Pro | 0.4 | Proposal only; researcher-reviewed |
| Blind benchmark | 400-review Human Reference benchmark | DeepSeek-V4-Pro | 0.0 | Blind to Human Reference labels |
| Full-corpus extension | 1,304-review AI-assisted pre-coding | DeepSeek-V4-Pro | 0.0 | Pre-coding only; 100% human verification required |
| Final labels | 400 + 1,304 final coding | Human governed | — | Human Reference + verified/audited/adjudicated labels |
| Audit-sampling preparation | Auxiliary sampling-status assignment for 28 DeepSeek failures | Human researcher | — | Manual sampling-field assignment only; no verifier labels or final analytic labels changed |

Note. The final full-corpus protocol requested model = deepseek-v4-pro, temperature = 0.0, thinking disabled, one independent request per review, no conversation history, and the frozen 39-code codebook. AI predictions were explicitly designated PRE-CODING, not final labels. In this public supplement, source-linked illustrative review wording embedded in the historical production prompt/codebook is omitted to avoid redistribution of third-party user-generated text; Section 3 preserves the operational code names, definitions, inclusion/exclusion criteria, and boundary rules used for coding.

# 2. Development-stage codebook prompts retained for reproducibility

The two prompts below document development-stage open-coding and semantic-consolidation proposals used within the 200-review codebook-development subset. Those 200 reviews were part of the later 1,304-review extension, not an additional sample, and were recoded under the frozen 39-code framework for final analysis. No evaluative-content screening prompt or screening stage was used in the reported study. All 1,704 reviews in the frozen corpus were retained for formal coding, with C0 representing reviews without substantive reception content. Prompt instructions are reproduced in faithful English translation for transparency; embedded example review text is omitted where necessary to avoid redistribution of third-party user-generated content. The production pre-coding prompt is documented separately in Section 2.3 under the same public-release principle.

## 2.1. Development-stage open (inductive) coding prompt

Function. For each review in the 200-review development subset, generate 2–5 open codes for any substantive reception content present, staying close to the reviewer’s wording while ignoring plot summary, reading-progress statements, and unrelated material. This was a codebook-development proposal stage, not a screening or exclusion stage. Issued to DeepSeek-V4-flash (temperature 0.3).

**System prompt**

You are a qualitative coding assistant for literary reception research, assisting with the initial coding stage of reflexive thematic analysis.

Your task is to generate open-code labels for Douban reader reviews. The number of labels should depend on the core evaluative dimensions actually present in the review: prefer fewer, well-supported labels rather than forcing additional ones (see the “Number” principle below). Most reviews should receive 2–4 labels, and even complex long reviews should receive no more than 5. Never code plot summary, reading-progress information, or irrelevant details.

\## Coding principles

1. **Stay close to the data**:
 Labels should use the reviewer’s own wording, or wording very close to it, rather than becoming overly abstract.
 ✓ Good: “I could hardly breathe while reading,” “it made me think of similar events in my own country,” “the translation is restrained and powerful”
 ✗ Poor (too abstract): “emotional response,” “cross-cultural association,” “translation evaluation”

2. **Descriptive rather than interpretive**:
 Describe what the review “says” or “does”; do not infer the reviewer’s latent motives or psychology.
 ✓ Good: “mentions being unfamiliar with Korean history”
 ✗ Poor: “reflects deficiencies in the reader’s educational background”

3. **Keep labels specific**:
 Each label should be a short phrase of 4–12 Chinese characters capturing one specific dimension.
 A review may involve multiple dimensions (e.g., bodily response, formal evaluation, historical understanding); assign one label to each dimension.

4. **Do not presuppose themes**:
 Do not use meta-language such as “X theme,” “X dimension,” or “X type.”

5. **Number: control strictly; do not pad the list**:
 Code only the **most central and prominent** evaluative dimensions in the review. Most reviews should receive 2–4 labels.
 - Very short reviews (e.g., “highly recommended,” “very oppressive”): 1–2 labels.
 - Ordinary reviews: 2–4 labels.
 - Even for a complex long review, **do not exceed 5 labels**—capture the most important dimensions and omit secondary ones.
 It is better to provide one fewer accurate label than one additional weakly justified label.

 **Never create a separate label for the following (they are noise, not evaluative dimensions):**
 - Reading-progress/count information, such as “my fifth Han Kang book,” “read it in two days,” or “second read”—these are not evaluations.
 - Passing, insignificant details, such as “opened it in winter” or “read it on the subway,” unless they are central to the review.
 - Plot summary, such as “after her friend was injured, she asked me to go to Jeju,” “the father was on a ventilator,” or “the bird was dying”—these retell the story rather than express the reader’s evaluation or response and must not be coded.
 - Self-check: if a review makes you want to assign more than five labels, stop and ask whether the later labels are genuinely independent evaluative dimensions or whether you are padding the list/repeating plot. If the latter, delete them.

\## Label format

- Each label should contain 4–12 Chinese characters.
- Use noun phrases or gerund-like phrases.
- Do not use punctuation.
- Do not use serial numbers or numbering.

\## Output format

Return strict JSON only, with no other text:

```json
{
 "codes": ["label 1", "label 2", "label 3"],
 "rationale": "Briefly explain the coding logic in one sentence (no more than 30 Chinese characters)."
}
```

**User-message template**

Generate open-code labels for the following review (use only as many dimensions as are actually present; prefer fewer well-supported labels). Return strict JSON only.

Review:
{comment_text}

## 2.2. Development-stage semantic-consolidation proposal prompt

Function. Propose candidate higher-level organizational groupings from semantic clusters, with rationale, confidence, and explicit uncertainty notes. This was a development-stage proposal only. The researchers reviewed, revised, accepted, split, merged, or discarded the proposals while constructing the coding frame. The historical prompt included a coverage instruction requiring every input cluster to appear in at least one candidate theme; that instruction is retained below in faithful English translation for transparency, but its candidate-theme output was not carried into the final analytic structure. The candidate “themes” named inside the historical prompt were not retained as final analytic themes; the final corpus-wide analysis uses 39 fixed binary codes summarized into eight predefined descriptive code groups. Issued to DeepSeek-V4-Pro (temperature 0.4).

**System prompt**

You are a qualitative-analysis assistant for literary reception research, assisting with the “searching for/reviewing themes” stage of reflexive thematic analysis.

The researchers have obtained a set of clusters (each cluster is a group of semantically similar open-code labels). Your task is to **propose** how these clusters could be consolidated into higher-level **candidate themes** for researchers to consider.

**Important:** You are making **proposals**, not final conclusions. The researchers will review, revise, or reject each proposal. Therefore:
- Provide a **rationale for consolidation** and a **confidence level** (high/medium/low) for each proposal.
- Proactively identify **uncertainties or points of dispute**: which clusters could reasonably be assigned elsewhere, which may need to be split, and which have blurred boundaries.
- Do not force all clusters into a small number of themes; it is preferable to retain a “for researcher decision” status where appropriate.

Possible perspectives for consolidation (use as guidance, not as a rigid template) include readers’ emotional/bodily responses, evaluation of literary form, historical and ethical understanding, reception stance (positive/negative), and cross-textual or cross-cultural associations.

\## Output format (strict JSON; no other text)
```json
{
 "candidate_themes": [
 {
 "theme_name": "Candidate theme name (8–20 Chinese characters)",
 "rationale": "Why these clusters are grouped together (30–80 Chinese characters)",
 "confidence": "high/medium/low",
 "member_clusters": ["cluster name 1", "cluster name 2"],
 "uncertainty_note": "Explanation of uncertainty/disagreement; leave blank if none"
 }
 ],
 "cross_cutting_notes": "Cross-theme observations: which clusters are hard to classify, which themes may overlap, and reminders for the researchers (50–150 Chinese characters)"
}
```
Requirement: every input cluster must appear in at least one candidate theme’s `member_clusters`.

**User-message template**

Below are {n} clusters (cluster name / description / total label frequency). Propose how they could be consolidated into candidate themes for researchers to consider.

Cluster list:
{cluster_list}

Return the proposal in strict JSON only. Remember: you are making recommendations, so provide a rationale, confidence level, and uncertainty notes.

## 2.3. Final 39-code production pre-coding prompt

Function. Apply the frozen 39-code codebook to one review as AI-assisted PRE-CODING. The production runner SHA256 is recorded below. Human Reference labels were not read by the runner. All 1,304 extension reviews subsequently underwent Human C verification: 1,276 valid AI pre-code outputs were checked and, when needed, modified; the 28 failed outputs were coded directly from review text. The public transcript below preserves the production instructions and JSON structure but omits source-linked illustrative review wording embedded in the frozen codebook; Section 3 provides the operational codebook fields used for public reproducibility.

Frozen production runner SHA256: 1dee5dd31d615e1b084688a1b5935d28fb0e976902de06e4557fa6b52689b6da

**System prompt**

You are performing AI-assisted PRE-CODING for a human-governed qualitative content analysis.
Your output is not the final analytic label. Every review will later undergo human verification.

Apply ONLY the frozen Codebook v1.0 reproduced below.
Do not invent, merge, rename, or reinterpret codes beyond the written rules.

GENERAL RULES
1. Unit: one reader review.
2. Assign every one of the 39 codes a binary label 0 or 1. Multi-label coding is allowed.
3. Distinguish the reviewer's own reception from quotations, plot summary, character states, or external factual statements.
4. C0 is mutually exclusive with all substantive codes:
 - if ANY substantive code C1.1-C8.5 = 1, then C0 MUST = 0;
 - if ALL substantive codes C1.1-C8.5 = 0, then C0 MUST = 1.
 An all-zero 39-code label vector is INVALID.
5. Before returning JSON, explicitly verify that exactly one of these two states holds:
 (A) C0=1 and all substantive codes=0; OR
 (B) C0=0 and at least one substantive code=1.
6. Use the Definition, Include when, Exclude when, Boundary rule, and examples exactly as frozen.
7. Even when uncertain, make a final 0/1 decision; list borderline decisions in uncertain_codes.
8. Return JSON only. Do not add prose outside JSON.
9. Do NOT return positive_codes. The runner will derive positive_codes mechanically from labels after validation.
10. coding_notes should contain brief evidence/reason only for codes that are positive or uncertain.
11. You have no access to human-reference labels or prior benchmark labels.

REQUIRED JSON SHAPE
{
 "analysis_id": "R0001",
 "labels": {"C0": 0, "C1.1": 0, "C1.2": 0, "C1.3": 0, "C1.4": 0, "C1.5": 0, "C1.6": 0, "C1.7": 0, "C2.1": 0, "C2.2": 0, "C2.3": 0, "C2.4": 0, "C2.5": 0, "C2.6": 0, "C2.7": 0, "C3.1": 0, "C3.2": 0, "C3.3": 0, "C3.4": 0, "C4.1": 0, "C4.2": 0, "C4.3": 0, "C5.1": 0, "C5.2": 0, "C5.3": 0, "C6.1": 0, "C6.2": 0, "C6.3": 0, "C6.4": 0, "C6.5": 0, "C6.6": 0, "C7.1": 0, "C7.2": 0, "C7.3": 0, "C8.1": 0, "C8.2": 0, "C8.3": 0, "C8.4": 0, "C8.5": 0},
 "confidence": {"C0": 0.95, "C1.1": 0.95, "C1.2": 0.95, "C1.3": 0.95, "C1.4": 0.95, "C1.5": 0.95, "C1.6": 0.95, "C1.7": 0.95, "C2.1": 0.95, "C2.2": 0.95, "C2.3": 0.95, "C2.4": 0.95, "C2.5": 0.95, "C2.6": 0.95, "C2.7": 0.95, "C3.1": 0.95, "C3.2": 0.95, "C3.3": 0.95, "C3.4": 0.95, "C4.1": 0.95, "C4.2": 0.95, "C4.3": 0.95, "C5.1": 0.95, "C5.2": 0.95, "C5.3": 0.95, "C6.1": 0.95, "C6.2": 0.95, "C6.3": 0.95, "C6.4": 0.95, "C6.5": 0.95, "C6.6": 0.95, "C7.1": 0.95, "C7.2": 0.95, "C7.3": 0.95, "C8.1": 0.95, "C8.2": 0.95, "C8.3": 0.95, "C8.4": 0.95, "C8.5": 0.95},
 "uncertain_codes": [],
 "coding_notes": {}
}

FROZEN CODEBOOK
================
[PUBLIC-RELEASE REDACTION: The frozen internal production runner supplied the complete codebook, including source-linked illustrative example wording. To avoid redistributing third-party user-generated text, the public supplement omits those source-linked example fields from this embedded prompt transcript. Section 3 reproduces the operational codebook fields used for coding: code IDs and names, definitions, inclusion criteria, exclusion criteria, and boundary rules. The runner SHA256 above records the frozen internal runner.]

**User-message template**

Pre-code the following single review using the frozen codebook. Return JSON only in the required schema.

analysis_id: {rid}
comment_text:
{comment_text}

*Note. The system-prompt excerpt above is based on the literal string returned by build_system_prompt() in the frozen production runner, but the public supplement omits source-linked illustrative review wording embedded in the original codebook to avoid redistributing third-party user-generated content. The runner SHA256 records the frozen internal runner. The user-message template is recovered from the same runner without sending an API request. The brace placeholder denotes the review text supplied in each independent request. Section 3 reproduces all operational code fields used for coding.*

# 3. Final frozen 39-code reception codebook

The final analytic codebook contains 39 mutually operationalized binary codes: C0 (no codable reception content) and 38 substantive codes C1.1–C8.5. C0 is a logical exclusivity state: C0 = 1 only when all substantive codes are 0. The public representation below preserves code IDs and names, definitions, inclusion criteria, exclusion criteria, and boundary rules. Source-linked illustrative example fields from the internal frozen codebook are not redistributed. The full-corpus protocol was frozen before the 1,304-review pre-coding run. For English-language publication compliance, the original Chinese-language code-name and operational fields are presented here in faithful English translation; code IDs, coding boundaries, and analytic decisions are unchanged.

**Final frozen codebook (39 binary codes)**

| **Code** | **Codename(EN)** | **Original codename (English translation)** | **Definition** | **Includewhen** | **Excludewhen** | **Boundaryrule** |
| --- | --- | --- | --- | --- | --- | --- |
| C0 | No codable reception content | No clearly codable reception content | The review contains no evaluation, emotion, self-reported experience, reading behavior, or interpretive proposition that can clearly be attributed to reader reception. | Only quotations from the work, plot summary, juxtaposed imagery, or external factual statements are present, with no expression of the reviewer’s own reception. | An explicit evaluation, experience, behavior, or interpretation is present, such as “I think,” “it made me,” “reading it felt,” “my impression,” or “I understand it as.” | Strong content alone does not automatically constitute reception; an identifiable reviewer stance or response is required. Highly condensed imagery is coded only when it clearly points to the reader’s impression. |
| C1.1 | Sadness / pain / grief | Sadness, pain, and grief | The reviewer explicitly expresses negative emotional experience such as sadness, pain, wanting to cry, grief, sorrow, or desolation. | Direct emotional expressions such as “it really hurt and made me want to cry” or “I felt very sad and desolate after reading.” | Only a character’s suffering, or the fact that the work depicts suffering, is described without stating the reader’s own feeling. | Prioritize the reader’s own emotion. If the review also interprets a character’s long-term trauma, C6.1 may be coded as well. |
| C1.2 | Heaviness / oppression / despair | Oppression, heaviness, darkness, and despair | The reviewer reports a sustained atmosphere-related feeling of oppression, heaviness, darkness, suffocation, despair, or similar affective burden. | Explicit statements such as “oppressive,” “heavy,” “always in darkness,” or “despairing.” | A description of the writing as “cold” or “dark” without a reader-emotion component; depending on context, this may fall under C2.1. | Distinguish from C1.1: C1.1 centers on sadness/grief, whereas this code centers on sustained atmosphere and psychological burden. The two may co-occur. |
| C1.3 | Other salient / complex emotions | Shock, unease, anger, and other salient/complex emotions | The reviewer expresses salient emotions such as shock, being shaken, unease, anger, powerlessness, conflicted hope, or pleasure that do not fit well under sadness or heaviness. | Expressions such as “I was shocked,” “shaking and unsettling,” “anger and rejection,” or “a powerless yet complicated sense of hope.” | Bodily experiences such as feeling cold, goosebumps, breathing changes, or sweating should be coded primarily as C1.4. | Code only emotions explicitly expressed by the reviewer; do not infer a specific emotional object from ambiguous wording. |
| C1.4 | Embodied reception | Embodied reading response | The reader expresses the impact of reading through bodily sensations or physiological reactions, such as coldness, freezing, trembling, goosebumps, cold sweat, difficulty breathing, or physical discomfort. | Clear references to the reader’s bodily-emotional state after reading or confronting historical content, including metaphorical bodily expressions. | Ice/snow in the work, a character’s bodily pain, or depictions of torture themselves; do not code these unless they are linked to the reader’s experience. | Metaphorical language may also be coded when the direction is clearly “work/history → reader’s embodied feeling.” |
| C1.5 | Affective resonance / empathy / psychological proximity | Affective resonance, sympathy, and psychological proximity | The reviewer explicitly reports an emotional connection, resonance, sympathy, or reduced psychological distance in relation to victims, historical places, or unfamiliar experience. | Examples include “it resonated with me,” “I can understand and sympathize more,” “I will feel heavy-hearted when I visit Jeju in the future,” or “reading in Chinese made the victims feel more familiar.” | Use C1.6 for immersion/presence without an emotional connection; use C1.7 when the reviewer explicitly reports inability to empathize, emotional distance, or difficulty becoming emotionally involved; use C4.3 for knowledge gain only. | C1.5 records only explicit resonance/psychological proximity. C1.5=0 does not imply emotional distance. Explicit negative distance or failed empathy must be coded as C1.7. |
| C1.6 | Reported immersion / sense of presence | Immersion, identification, and sense of presence | The reviewer explicitly self-reports being drawn into the narrative world, entering a concrete scene, or experiencing a sense of presence. This code emphasizes subjective entry into a scene or narrative world, rather than general emotional intensity or bodily metaphor. | Explicit statements such as “I felt I was there,” “as if I were at the scene,” “I was suddenly pulled into the scene/story,” or “it felt as though I entered the space where the characters were.” The reviewer’s own report of entering the scene/narrative world is required. | “Immersed in sadness/despair” should receive the corresponding C1.* emotion code only. Expressions such as “right in front of me,” “enveloped,” “suffocating,” or “falling into the deep sea” that function only as bodily/effect metaphors should use C1.4/C2.3 as appropriate and do not automatically count as C1.6. Explicit difficulty identifying with the work uses C1.7. | The minimum evidence for C1.6 is an explicit sense of entering a scene/narrative world or being present there. The word “immersed” alone is insufficient; its object matters: immersion in emotion ≠ immersion in the narrative world. |
| C1.7 | Affective distance / difficulty empathizing or identifying | Emotional distance or difficulty empathizing/identifying | The reviewer explicitly reports psychological/emotional distance from characters, victims, the narrative, or the work, or explicitly states difficulty/inability to empathize, identify, or become emotionally involved. | Clear negative reception outcomes such as “I always feel a sense of distance,” “it is difficult to enter into it and invest emotion or empathy,” or “I cannot empathize.” If a cause is also stated, this code may co-occur with historical-knowledge, formal, or translation-barrier codes. | Do not infer distance simply because resonance/immersion is absent. General incomprehension without reference to emotional distance should use C3.*. Distance among characters within the work does not count. | Only record an explicit negative relational reception outcome; do not infer distance from C1.5/C1.6=0. Expressions such as “unable to empathize,” “it is difficult for it to move me,” or “I feel distance” qualify. “I cannot get into it” by itself should use C8.4/C3.* if it mainly indicates avoidance or comprehension difficulty rather than empathy/psychological distance. |
| C2.1 | Language / style appreciation or perception | Aesthetic evaluation of language, prose, and style | The reviewer makes an aesthetic evaluation of, or clearly perceives, the language, prose, sentences, brushwork, descriptive method, or verbal style, usually in positive or neutral terms. | Explicit evaluations such as “the language/prose/sentences/wording/brushwork/descriptions are delicate, austere, beautiful,” where the object is clearly language or descriptive method. | Use C2.5 for strongly negative evaluations of prose/literary expression; C5.1 for evaluation of translated wording; C8.1 for only broad evaluations of the story/book as good, bad, light, or heavy. | First identify the object of evaluation: language/sentences/descriptive method → C2.1; negative evaluation of a specific narrative/literary form → C2.5; broad evaluation of the book/story as a whole → C8.1. Do not infer C2.1 simply because the review itself is written poetically. |
| C2.2 | Narrative / formal appreciation or perception | Evaluation/perception of narrative form, structure, and viewpoint | The reviewer makes a neutral or positive evaluation, perception, or analysis of person, viewpoint, multi-character structure, conception, oral-history-like or visual presentation, stream of consciousness, structural organization, or other narrative form, where the focus is not a negative formal judgment itself. | Neutral/positive observations such as “multiple viewpoints form a panorama,” “I like the shifts in person,” “an oral-history style,” or “the different parts of the structure each do their job.” | If the same statement clearly criticizes the form as “fragmented, affected, showy, unsuccessful, too deliberate,” prioritize C2.5 and do not add C2.2 merely because form is mentioned. If form clearly causes difficulty, use C3.*. | C2.2 may co-occur with C2.5/C3.*, but only when there is independent evidence of neutral/positive formal observation or analysis. Do not use C2.2 to duplicate the same negative formal evaluation already captured by C2.5. |
| C2.3 | Imagery, title, symbolic meaning and visualization | Imagery, title, symbolic meaning, and visualized experience | A specific image, scene, title, symbolic detail, or metaphor leaves a clear impression on the reader, is liked/interpreted, or triggers vivid visualization and meaning-making. | Reception directed at a specific image/title/symbol, such as “the birds and rain left a strong impression,” “I really like the scene descriptions,” “‘We Do Not Part’ has three meanings to me,” or “the title suggests a hopeful future.” | Merely listing images/titles without a reception marker. Broad “theme interpretation” not anchored in a specific title, image, or symbol should not use this code; instead assess the relevant C6 historical/ethical/trauma code. | This is not a catch-all “theme interpretation” code. A specific carrier (image, title, scene, symbolic detail) is required; broader ethical/political/historical meaning remains under C6.*. |
| C2.4 | Appraisal of trauma / violence representation | Evaluation of how trauma and violence are represented | The reviewer evaluates how the author writes violence, pain, or trauma, including restraint, avoidance of sensationalism, emotionalization, individualization, exaggeration, or manipulation of empathy. | Examples include “violence is written with restraint,” “the author depicts pain very effectively,” “pain is exaggerated to solicit empathy,” or “history is written through personal experience.” | Anger at or reflection on historical violence itself should use C6.3/C6.4; the reader’s own pain should use C1.*. | The object of evaluation must be the **mode of representation**, not the violent event itself. |
| C2.5 | Negative formal / literary evaluation | Negative evaluation of literary expression and form | The reviewer explicitly evaluates the work’s style, literary expression, narrative technique, or related formal feature negatively, e.g., as contrived, affected, childish, issue-driven, or not to their taste. | Explicit criticism of narrative, prose, literary expression, or form, such as “the narration is too deliberate,” “too lyrical, like prose,” “rambling,” or “the style is affected.” | If the reviewer only says the whole book/story/other major part is poor or average without identifying a formal object, use C8.1. If form causes comprehension difficulty, add C3.*. Translation problems use C5. | Do not automatically assign C2.5 whenever the review says “poorly written.” Use it only when the object is identifiable as prose style, narrative technique, structure, form, or literary expression; otherwise use C8.1. |
| C2.6 | Form-over-content / historical adequacy criticism | Form outweighing content / inadequate historical representation | The reviewer believes poeticity, symbolism, lyricism, private emotion, or formal technique outweighs substantive content, historical fact, documentary value, or seriousness. | Examples include “poetry outweighs substantive content,” “emotion obscures the truth,” “symbolism overwhelms historicity,” or “historical reconstruction is limited.” | General dislike of the form uses C2.5; claims that the subject would be better handled as nonfiction/oral history use C2.7. | This code concerns a judgment about the relative weight of “form versus history/content” and does not require the reviewer to reject literariness itself. |
| C2.7 | Genre / medium suitability judgment | Judgment of genre/medium suitability | The reviewer judges that a historical subject is more or less suitable for a specific genre or medium, such as fiction, oral history, or nonfiction. | Examples include “it would be better as oral history” or “this subject is better suited to nonfiction.” | If the review only says the work “resembles oral history” without judging suitability, use C2.2. | This is the reader’s value judgment about mode of presentation, not an objective factual claim that the subject should use a particular genre. |
| C3.1 | General reading / comprehension difficulty | General reading or comprehension difficulty | The reviewer explicitly says the work is difficult to read, hard to understand, or hard to enter, but there is insufficient evidence to identify a specific cause. | Examples include “difficult to read,” “I couldn’t understand it,” “I didn’t really get it,” or “it was a little hard to get into at first.” | If the reviewer clearly attributes the difficulty to historical background, translation, fragmentation, person/viewpoint, chronology, or similar factors, use the corresponding specific code. | Do not infer the source of difficulty on the researcher’s behalf; when the cause is unclear, remain at general difficulty. |
| C3.2 | Fragmentation / abstraction / jumpiness difficulty | Difficulty caused by fragmentation, abstraction, or jumpiness | Formal features such as fragmentation, stream of consciousness, dreamlike writing, abstraction, jumpiness, disorder, convolutedness, or general blurring of reality and unreality cause distraction, disengagement, difficulty reading, disorientation, difficulty identifying the main point, or difficulty entering the text. | The review explicitly links fragmentation, stream of consciousness, abstraction, jumpiness, dreamlike/disordered writing, or “reality and unreality intertwined / convolutedness” to difficulty, fatigue, disorientation, disengagement, difficulty identifying the main point, or comprehension problems. | Do not code C3.2 if the review merely says “very jumpy/fragmented/stream-of-consciousness” without a difficulty outcome. Use C3.3 for explicit person-identification difficulty; use C3.4 when the difficulty is explicitly caused by transitions in time, scene, or reality–dream states. | General fragmentation/stream of consciousness/abstraction/jumpiness → difficulty is primarily C3.2. Do not infer C3.4 merely from words such as “reality/unreality,” “dream,” or “nonlinear”; C3.4 requires an explicit statement that a specific transition/switching mechanism causes difficulty. |
| C3.3 | Person / viewpoint / subject-identification difficulty | Difficulty identifying person, viewpoint, or subject | Shifts in person or viewpoint make it difficult for the reviewer to identify the speaker, grammatical subject, or character position. | Examples include “every time the scene changes I have to guess the subject” or “the shifts in person make it hard to tell who is speaking.” | Merely noticing or liking multiple viewpoints/persons without difficulty should use C2.2. | Do not code automatically because the work objectively uses multiple viewpoints; evidence of reader difficulty is required. |
| C3.4 | Temporal / scene / reality-imagination transition difficulty | Difficulty with temporal, scene, or reality/imagination transitions | The reviewer explicitly states that transitions in time sequence, scene, or between reality and dream/hallucination/imagination themselves cause difficulty with recognition, orientation, or comprehension. | Examples include “the constant switching between reality and dreams makes it hard to tell them apart,” “the scene and time shifts keep making it hard to understand,” or “every scene change makes me re-establish the time point.” | If the review only says “stream of consciousness/nonlinear/reality and unreality intertwined/many dreams” and finds the work difficult, but does not explicitly identify a transition mechanism, prioritize C3.2. If there is only formal perception without difficulty, use C2.2. | C3.4 requires both: (1) an explicit transition in time/scene/reality–dream states and (2) an explicit attribution of difficulty to that transition. Related imagery or the mere phrase “reality/unreality” is insufficient. |
| C4.1 | Historical knowledge barrier | Insufficient historical background knowledge / contextual barrier | The reviewer explicitly reports difficulty understanding, entering, or locating the work because of insufficient knowledge of the relevant historical, political, or social background. | Examples include “I don’t know this history, so I can’t get into it” or “if you are unfamiliar with the background, it feels jumpy.” | Use C3.1 for general incomprehension without a historical cause; use C5.2 for difficulty caused by translation. | Code only when the reviewer explicitly attributes the difficulty to background knowledge; do not infer the cause. If the reviewer further states that this makes empathy/identification difficult or creates emotional distance, C1.7 may also be coded. |
| C4.2 | Active historical contextualization | Active consultation/supplementation of historical background | The reviewer actively consults encyclopedias, reference materials, or other sources before, during, or after reading to supplement the historical background of the work. | Examples include “I looked up the historical background before and after reading” or “I copied a passage from an encyclopedia.” | If the reviewer only says they do not know the history but does not report any supplementary action, use C4.1. | The act of active searching itself is sufficient; it is not necessary to show that the search led to deeper understanding. |
| C4.3 | New historical awareness / educational value | New historical awareness, knowledge gain, and educational value | Reading produces new historical understanding, surprise at previously unknown events, or a belief that the work helps readers understand unfamiliar history and fills an educational/cognitive gap. | Examples include “I had no idea this happened on Jeju” or “it filled a gap in my education about unfamiliar history and sympathetic understanding.” | Use C4.2 for looking up information without stating new understanding; use C4.1 when lack of background knowledge causes difficulty. | Continue to record new historical awareness/educational value produced by reading. If future formal samples contain an explicit negative historical-learning effect, retain it under this code and first consider adding a valence auxiliary variable (positive/negative) rather than immediately splitting the code. |
| C5.1 | Translation evaluation | Evaluation of translation quality | The reviewer makes a positive or negative evaluation of the Chinese translation, wording, syntax, or translation quality. | Examples include “the translation is very poor,” “some words need reconsideration,” or “the translated long modifiers read like long English sentences.” | Evaluation of Han Kang’s original language/style uses C2.1/C2.5. | If a translation problem is also explicitly said to make the text difficult to understand, code C5.2 as well. |
| C5.2 | Translation-induced difficulty | Translation-induced reading/comprehension difficulty | The reviewer explicitly states that the syntax, wording, or quality of the Chinese translation makes reading more difficult, convoluted, hard to understand, or close to being abandoned. | Examples include “a hell of modifiers,” “the sentences are too long and winding,” or “the translation was so bad I almost gave up in the first few chapters.” | If the reviewer only says “the translation is poor” without a reading barrier, use C5.1 only; difficulty caused by the original work’s form uses C3.*. | There must be an identifiable “translation/translated text → reading difficulty” relationship. |
| C5.3 | Paratext / edition design evaluation | Evaluation of paratext and edition design | The reviewer explicitly evaluates how a preface, foreword, contents page, cover/binding, layout, chapter arrangement, or other edition/paratextual feature affects reading experience, expectations, or overall evaluation. | Examples include “going directly into the text with no preface or contents page is a major plus” or “the cover/design shaped my reading expectations.” | Issues of Chinese translation quality/syntax/word choice use C5.1/C5.2; narrative structure in the main text uses C2.2/C3.*; merely identifying which edition was read without evaluation is not coded. | The object must be a presentation/organizational feature outside the main text or at the edition level. If paratext also affects prior expectations, C8.2 may co-occur. |
| C6.1 | Understanding of long-term / transgenerational trauma | Understanding victims’/survivors’ long-term or transgenerational trauma | The reviewer uses their own summary or interpretation to understand continuing physical, psychological, social, or transgenerational trauma experienced by victims, survivors, and their families after the event. | Examples include “those who survived could not recover for a long time,” “the shadow of war hangs over several generations,” or “survivor guilt.” | Do not code if the review only reproduces character statements, plot, or trauma scenes without the reviewer’s own synthesis. | Interpretive synthesis of characters’ trauma counts as reader meaning-making; plot summary does not. |
| C6.2 | Memory / non-forgetting / retelling ethics | Ethics of memory, non-forgetting, and retelling | The reviewer treats remembering, mourning, telling, refusing to forget, not saying goodbye, or preventing recurrence as an ethical imperative. | Examples include “writing it down is a way to remember,” “history should be told,” “do not forget even when it hurts,” or “everyone should know.” | If the review only says the work contains a memory theme without endorsing or evaluating it, use C6.5 depending on context or do not code. | This may be reception of a theme or the reviewer’s own normative stance; an explicit phrase such as “I think” is not required. |
| C6.3 | State violence / responsibility / justice reflection | Reflection on state violence, responsibility, justice, and revenge | The reviewer, in their own judgment/reflection or explicit endorsement, discusses state violence, institutional responsibility, structures of power/perpetration, revenge, justice, censorship/political repression, or narratives of political loyalty. | Examples include the reviewer explicitly asking or judging “how should the state/institutions be held responsible,” “would I still become numb as a media worker,” or “self-censorship caused by censorship,” or explicitly endorsing a related political proposition in the work. | A standalone political question/character line quoted from the novel, external historical material, or political fact does not automatically qualify without the reviewer’s own judgment/endorsement. Pure slogans unrelated to the work also do not automatically qualify. | First determine whose political proposition it is: it must be attributable to the reviewer, or have explicit endorsement/continuation by the reviewer. Unguided quotations, excerpts, or informational statements should not be coded C6.3 merely because their political content is strong. |
| C6.4 | Human nature / violence / dignity / existential reflection | Reflection on human nature, violence, dignity, existence, and peace | The reviewer extends the work into reflection on human cruelty/courage, human dignity, capacity to harm, bystander responsibility, the fragility of peace, or individual existence. | Examples include “why are people both cruel and brave,” “we are not meat,” “what humans can do to others,” or “people only regret peace after losing it.” | If the main focus is state/institutional responsibility, use C6.3; if the main focus is remembering/telling, use C6.2. | The reflection may extend from the work to the reviewer’s own life or a general human condition, as long as the relationship to reading is identifiable. |
| C6.5 | Literature as memory / witness / historical writing | Value of literature as memory, witness, and historical writing | The reviewer affirms or discusses literature’s ability and value in preserving memory, witnessing trauma, reconstructing history, making the dead visible, or assuming responsibility through language. | Examples include “literature can sometimes outlast history,” “using literature to reconstruct traumatic memory,” or “I admire the courage to confront history in writing.” | If the review only insists “do not forget” without discussing the role of literature/writing, use C6.2. | Authorial courage may be included when it is clearly tied to the value of writing/witnessing historical trauma rather than general admiration for the author. |
| C6.6 | Reader attribution/evaluation of author intent, position, or persona | Reader attribution/evaluation of authorial intention, position, or persona | The reviewer speculates about, attributes, or evaluates the author’s writing intention, motivation, emotion, stance, historical position/distance, or the persona/image/temperament presented as a writer. | Examples include “is the author trying to draw attention to state violence,” “she must have been in pain while writing,” “the expression of nihilism may come from distance from the event,” or “as a writer she seems narcissistic/strong/noble.” | Do not code reliable external factual statements that are not reader interpretation; do not use this code for “I like/dislike Han Kang” alone when there is no content about intention, position, or writerly persona. | All such judgments must be reported in the paper as “the reviewer believes/speculates/evaluates that the author…” and must never be restated as facts about Han Kang’s actual psychology, personality, or intention. Authorial courage in historical writing/witnessing may co-occur with C6.5. |
| C7.1 | Comparison with Han Kang's other works | Comparison with Han Kang’s other works | The reviewer compares the focal work with Han Kang’s other works or uses experience of earlier works to position the current reading. | Examples include “better than The Vegetarian,” “similar in conception to The Vegetarian,” or “the earlier work was too abstract, so I chose this one.” | Comparisons with works/films by other creators use C7.2. | Comparison may occur as a pre-reading motivation or as a post-reading aesthetic/value reassessment and may co-occur with C8.2 or C8.1. |
| C7.2 | Intertextual comparison with other works / media | Intertextual comparison with other works, film/TV, or media | The reviewer links, compares, contrasts, or accumulates experience between the focal work and a specific work by another author, oral history, film, documentary, or an explicit category of work/media. | Examples include “it reminded me of Moses on the Plain,” “it feels like Yu Hua’s The Seventh Day,” “compared with documentaries,” or “there are already many film/TV and literary works on the same subject.” | Comparison with Han Kang’s own works uses C7.1; association with China/the reviewer’s own historical reality uses C7.3. | The comparison target should preferably be identifiable as a specific work or media category. Even without a specific title, an explicit external literary/film/media reference used to understand or evaluate the work may be coded. A vague statement such as “there is a lot of media coverage” without a comparative function is not coded. |
| C7.3 | Cross-cultural / cross-historical association and world-literary positioning | Cross-cultural/cross-historical association and world-literary positioning | The reviewer explicitly connects, compares, or positions the work in relation to another country/culture, another historical situation, the reviewer’s own social history, or an East Asian/Asian/world-literary political context. | Examples include “Korean and Chinese-language literatures write suffering differently,” “this made me think of our own history,” “the horror is comparable to the Japanese invasion,” or “I see Han Kang in an East Asian/world-literary context.” | Use C7.2 for comparison with a specific book/film; use C4.* for simply looking up history. Do not code C7.3 for vague expressions such as “it feels familiar” or “Korean literature/film has a sense of mission” without an explicit second cultural/historical referent. | The minimum evidence is an explicit cross-national, cross-historical, cross-cultural, or world-literary positioning relationship. Do not supply a comparison target on the researcher’s behalf when wording such as “familiar,” “shared by humanity,” or “we” is too ambiguous. |
| C8.1 | Overall evaluation / value judgment | Overall evaluation and value judgment | The reviewer makes an explicit judgment of good/bad, like/dislike, worth/not worth, a rating, or a broad comparative value judgment about the work as a whole, the story as a whole, a major portion, or its overall value. | Examples include “a good book,” “overall it was pretty good,” “personally I don’t like this book/this overall approach and subject,” “3.5 stars,” or “overall this book is not as good as the earlier one.” | Do not code C8.1 for only overall feelings/process descriptions such as “strange,” “heavy,” “tiring,” or “special” without an explicit value judgment. Specific evaluations of language/form/translation should use the corresponding detailed code and should not automatically be upgraded to an overall evaluation. | First confirm both scope and evaluative valence: C8.1 requires an explicit judgment of good/bad, like/dislike, worth, rating, or broad comparison directed at the whole book/story/major portion. Independent specific evaluations of form/language may co-occur with detailed codes. |
| C8.2 | Reading motivation / expectation | Reading motivation and prior expectation | The reviewer explains why they chose to read the book or describes pre-reading interest/expectations, including motivation from the Nobel Prize, author prestige, prior works, or interest in the subject. | Examples include “I read it because of the Nobel Prize,” “I was curious about the Gwangju subject and its literary presentation,” or “the earlier work was too abstract, so I chose this one.” | Use C8.4 for post-reading intentions to reread or continue trying to understand; use C8.1 for overall evaluation of the work. | Reading motivation may co-occur with comparison across works, author prestige, and related factors; do not treat motivation itself as an outcome of reception. |
| C8.3 | Reading attraction, boredom, fatigue, and retention | Reading attraction, boredom/fatigue, and textual retention | The reviewer evaluates how engaging or smooth the reading process was, reports boredom, fatigue, distraction, or how much content/plot was retained after reading; this also includes thematic/aesthetic fatigue from repeated exposure to the same historical subject or its representations across media. | Explicit statements that the text was engaging, smooth/not smooth, boring, tiring, distracting, or left almost no retained content after reading; or explicit “aesthetic fatigue” from repeated literary/film/TV treatment of the same historical subject. | If the review only says “I finished it/kept reading/stopped midway/skipped passages” without evaluating the quality of the reading process, use C8.4. Use C3.1 for general incomprehension and specific C3/C4/C5 codes for identified barriers. Do not code merely because the subject is common unless fatigue is expressed. | Explicit reading/aesthetic fatigue caused by repeated exposure to the same historical subject or cross-media representations is also coded C8.3. If the review also clearly links other literary/film/media works, C7.2 may co-occur. Completion behavior itself remains primarily C8.4. |
| C8.4 | Reading behavior / coping strategy | Reading behavior and coping strategy | The reviewer describes concrete reading behaviors or strategies such as persisting to the end, considering abandonment, pausing, changing environment, rereading, close reading, or continuing to work toward understanding. | Examples include “I couldn’t help stopping,” “I read on the subway to buffer the emotion,” “I didn’t understand it but I want to,” or “I will take it out again and read it carefully.” | If the review only says the work is difficult/boring without a behavioral consequence, use C3.1/C8.3 respectively. | The behavior and its cause may be coded separately. If “I got through it/finished it/finally finished it” primarily emphasizes completion behavior, prioritize this code; add C8.3 only when there is an explicit judgment about process quality such as smoothness, attraction, or boredom. |
| C8.5 | Meta-reception / evaluation frames | Meta-reception and evaluative frames | The reviewer reflects on how they themselves should evaluate, read, or discuss the work, or explicitly evaluates/explains how other readers, rating groups, or social audiences evaluate the work and why. | Examples include “I don’t want to evaluate her books in the manner of literary criticism” or “many people who think Han Kang is good are using the ideas of a particular historical moment for self-expression”—clear reflection/attribution about one’s own or others’ evaluative frames. | Ordinary literary evaluation, overall like/dislike, or simply reporting a Douban score or saying “many people gave five stars” without explaining the evaluative method/reason does not use this code. | This code records second-order commentary on the act of evaluation itself and may concern the reviewer or other readers/groups. Do not use it to prematurely infer fixed reader types or theoretical binaries. |

***Note.*** *The codebook was frozen before the formal 1,304-review extension run. For downstream analysis, all 1,704 reviews were represented exactly once in the final 39-code matrix.*

# 4. Human reliability, benchmark validation, and audit

Reliability was evaluated before adjudication where appropriate. Adjudication was then used to construct final reference or audit labels; post-adjudication human–human reliability was not recalculated.

**Supplementary Table S2. Pre-adjudication human–human reliability for the 400-review validation sample**

| **Code** | **N** | **Raw agreement** | **Cohen’s κ** | **Bootstrap 95% CI for κ** | **Positive agreement** | **Negative agreement** |
| --- | --- | --- | --- | --- | --- | --- |
| C0 | 400 | 92.5% | 0.628 | 0.501–0.745 | 66.7% | 95.8% |
| C1.1 | 400 | 91.8% | 0.719 | 0.625–0.804 | 76.9% | 95.0% |
| C1.2 | 400 | 92.2% | 0.625 | 0.499–0.740 | 66.7% | 95.6% |
| C1.3 | 400 | 89.0% | 0.427 | 0.285–0.557 | 47.6% | 93.9% |
| C1.4 | 400 | 94.0% | 0.615 | 0.463–0.745 | 64.7% | 96.7% |
| C1.5 | 400 | 95.5% | 0.630 | 0.447–0.777 | 65.4% | 97.6% |
| C1.6 | 400 | 96.5% | 0.444 | 0.159–0.662 | 46.2% | 98.2% |
| C1.7 | 400 | 98.5% | 0.720 | 0.453–0.907 | 72.7% | 99.2% |
| C2.1 | 400 | 90.8% | 0.726 | 0.639–0.804 | 78.4% | 94.1% |
| C2.2 | 400 | 93.0% | 0.771 | 0.683–0.847 | 81.3% | 95.7% |
| C2.3 | 400 | 90.5% | 0.552 | 0.417–0.671 | 60.4% | 94.6% |
| C2.4 | 400 | 86.5% | 0.554 | 0.446–0.655 | 63.5% | 91.7% |
| C2.5 | 400 | 92.2% | 0.735 | 0.644–0.819 | 78.0% | 95.3% |
| C2.6 | 400 | 97.0% | 0.698 | 0.510–0.842 | 71.4% | 98.4% |
| C2.7 | 400 | 99.2% | 0.568 | 0.000–1.000 | 57.1% | 99.6% |
| C3.1 | 400 | 95.5% | 0.666 | 0.502–0.797 | 69.0% | 97.6% |
| C3.2 | 400 | 96.5% | 0.399 | 0.110–0.630 | 41.7% | 98.2% |
| C3.3 | 400 | 97.5% | 0.362 | -0.011–0.660 | 37.5% | 98.7% |
| C3.4 | 400 | 99.0% | 0.595 | -0.003–0.908 | 60.0% | 99.5% |
| C4.1 | 400 | 98.8% | 0.438 | -0.008–0.799 | 44.4% | 99.4% |
| C4.2 | 400 | 98.8% | 0.540 | -0.004–0.856 | 54.5% | 99.4% |
| C4.3 | 400 | 96.8% | 0.564 | 0.320–0.757 | 58.1% | 98.3% |
| C5.1 | 400 | 98.2% | 0.910 | 0.834–0.969 | 92.0% | 99.0% |
| C5.2 | 400 | 99.0% | 0.813 | 0.579–0.962 | 81.8% | 99.5% |
| C5.3 | 400 | 99.5% | 0.664 | 0.000–1.000 | 66.7% | 99.7% |
| C6.1 | 400 | 96.0% | 0.839 | 0.754–0.910 | 86.2% | 97.7% |
| C6.2 | 400 | 94.2% | 0.773 | 0.680–0.856 | 80.7% | 96.6% |
| C6.3 | 400 | 87.5% | 0.518 | 0.394–0.628 | 59.0% | 92.6% |
| C6.4 | 400 | 90.5% | 0.620 | 0.504–0.722 | 67.2% | 94.4% |
| C6.5 | 400 | 94.5% | 0.688 | 0.551–0.802 | 71.8% | 97.0% |
| C6.6 | 400 | 88.8% | 0.552 | 0.433–0.659 | 60.9% | 93.4% |
| C7.1 | 400 | 95.2% | 0.781 | 0.678–0.869 | 80.8% | 97.3% |
| C7.2 | 400 | 94.0% | 0.700 | 0.576–0.804 | 73.3% | 96.6% |
| C7.3 | 400 | 94.5% | 0.563 | 0.382–0.712 | 59.3% | 97.1% |
| C8.1 | 400 | 88.2% | 0.704 | 0.625–0.779 | 78.3% | 91.9% |
| C8.2 | 400 | 96.0% | 0.481 | 0.231–0.683 | 50.0% | 97.9% |
| C8.3 | 400 | 91.0% | 0.464 | 0.302–0.605 | 51.4% | 95.0% |
| C8.4 | 400 | 93.2% | 0.639 | 0.504–0.758 | 67.5% | 96.2% |
| C8.5 | 400 | 94.2% | 0.245 | 0.060–0.442 | 25.8% | 97.0% |

***Note.*** *Two human coders independently coded the same 400 reviews under the frozen 39-code system. Paired nonparametric percentile bootstrap confidence intervals used 10,000 review-level resamples with coder pairs preserved (seed 20260814). These are pre-adjudication estimates.*

**Supplementary Table S2A. Per-code 2 × 2 agreement-cell counts for the two independent human coders**

| **Code** | **Code name** | **A=0, B=0** | **A=0, B=1** | **A=1, B=0** | **A=1, B=1** |
| --- | --- | --- | --- | --- | --- |
| C0 | No codable reception content | 340 | 28 | 2 | 30 |
| C1.1 | Sadness / pain / grief | 312 | 14 | 19 | 55 |
| C1.2 | Heaviness / oppression / despair | 338 | 25 | 6 | 31 |
| C1.3 | Other salient / complex emotions | 336 | 4 | 40 | 20 |
| C1.4 | Embodied reception | 354 | 6 | 18 | 22 |
| C1.5 | Affective resonance / empathy / psychological proximity | 365 | 10 | 8 | 17 |
| C1.6 | Reported immersion / sense of presence | 380 | 6 | 8 | 6 |
| C1.7 | Affective distance / difficulty empathizing or identifying | 386 | 2 | 4 | 8 |
| C2.1 | Language / style appreciation or perception | 296 | 28 | 9 | 67 |
| C2.2 | Narrative / formal appreciation or perception | 311 | 7 | 21 | 61 |
| C2.3 | Imagery, title, symbolic meaning and visualization | 333 | 11 | 27 | 29 |
| C2.4 | Appraisal of trauma / violence representation | 299 | 17 | 37 | 47 |
| C2.5 | Negative formal / literary evaluation | 314 | 4 | 27 | 55 |
| C2.6 | Form-over-content / historical adequacy criticism | 373 | 5 | 7 | 15 |
| C2.7 | Genre / medium suitability judgment | 395 | 0 | 3 | 2 |
| C3.1 | General reading / comprehension difficulty | 362 | 12 | 6 | 20 |
| C3.2 | Fragmentation / abstraction / jumpiness difficulty | 381 | 7 | 7 | 5 |
| C3.3 | Person / viewpoint / subject-identification difficulty | 387 | 5 | 5 | 3 |
| C3.4 | Temporal / scene / reality-imagination transition difficulty | 393 | 3 | 1 | 3 |
| C4.1 | Historical knowledge barrier | 393 | 3 | 2 | 2 |
| C4.2 | Active historical contextualization | 392 | 1 | 4 | 3 |
| C4.3 | New historical awareness / educational value | 378 | 8 | 5 | 9 |
| C5.1 | Translation evaluation | 353 | 5 | 2 | 40 |
| C5.2 | Translation-induced difficulty | 387 | 1 | 3 | 9 |
| C5.3 | Paratext / edition design evaluation | 396 | 0 | 2 | 2 |
| C6.1 | Understanding of long-term / transgenerational trauma | 334 | 9 | 7 | 50 |
| C6.2 | Memory / non-forgetting / retelling ethics | 329 | 17 | 6 | 48 |
| C6.3 | State violence / responsibility / justice reflection | 314 | 32 | 18 | 36 |
| C6.4 | Human nature / violence / dignity / existential reflection | 323 | 7 | 31 | 39 |
| C6.5 | Literature as memory / witness / historical writing | 350 | 13 | 9 | 28 |
| C6.6 | Reader attribution/evaluation of author intent, position, or persona | 320 | 3 | 42 | 35 |
| C7.1 | Comparison with Han Kang's other works | 341 | 7 | 12 | 40 |
| C7.2 | Intertextual comparison with other works / media | 343 | 6 | 18 | 33 |
| C7.3 | Cross-cultural / cross-historical association and world-literary positioning | 362 | 8 | 14 | 16 |
| C8.1 | Overall evaluation / value judgment | 268 | 37 | 10 | 85 |
| C8.2 | Reading motivation / expectation | 376 | 4 | 12 | 8 |
| C8.3 | Reading attraction, boredom, fatigue, and retention | 345 | 19 | 17 | 19 |
| C8.4 | Reading behavior / coping strategy | 345 | 4 | 23 | 28 |
| C8.5 | Meta-reception / evaluation frames | 373 | 0 | 23 | 4 |

Note. These cell counts are shown to make the direction and frequency of human-coder disagreements transparent. They are pre-adjudication counts. Neither coder is treated as a gold standard in this table.

**Supplementary Table S3. Sampling and construction of the adjudicated 400-review Human Reference set**

| **Index** | **Value** | **Interpretation** |
| --- | --- | --- |
| Development subset excluded from validation pool | 200 reviews (100 per novel) | Codebook-development reviews were excluded before validation sampling |
| Remaining validation pool | 1,504 reviews | 1,704 frozen-corpus reviews − 200 development reviews |
| Validation sampling rule | 200 reviews per novel | Stratified random sampling without replacement |
| Overlap with development subset | 0 | PASS; the 400-review Human Reference was separate from codebook development |
| Human Reference reviews | 400 | Locked adjudicated human reference set |
| Binary code decisions | 15,600 | 400 reviews × 39 codes |
| Coder A/B agreements inherited | 14,707 | Common human decision retained |
| Third-human adjudications used | 893 | Used only where A ≠ B |
| Reviews containing ≥1 adjudication | 299 | Out of 400 reference reviews |
| C0 logic conflicts | 0 | Final reference set passed exclusivity QC |

***Note.*** *Reference-standard rule: if Coder A = Coder B, the common human decision was inherited; if A ≠ B, the third independent human adjudicator’s final decision was used. Human–human reliability was not recalculated after adjudication.*

**Supplementary Table S4. Blind DeepSeek-V4-Pro benchmark against the adjudicated Human Reference**

| **Run** | **Valid N** | **Micro precision** | **Micro recall** | **Micro F1** | **Macro F1** | **Mean raw agreement** | **Mean code κ** | **Exact-match accuracy** | **Mean Jaccard** |
| --- | --- | --- | --- | --- | --- | --- | --- | --- | --- |
| RUN1 | 397 | 0.856 | 0.705 | 0.773 | 0.763 | 0.956 | 0.739 | 0.312 | 0.670 |
| RUN2 | 396 | 0.861 | 0.711 | 0.779 | 0.762 | 0.957 | 0.739 | 0.328 | 0.680 |

***Note.*** *RUN1 had 3 technical FAILED rows and RUN2 had 4; FAILED rows were not imputed, manually repaired, or assigned C0. Each run was evaluated only on its valid rows. The model was blind to final Human Reference labels.*

**Supplementary Table S5. Frozen full-corpus 39-code production protocol**

| **Component** | **Frozen rule** |
| --- | --- |
| Analytic universe | 1,704 reviews; frozen 39-code codebook |
| Human Reference | 400 adjudicated human reviews, sampled as 200 per novel without replacement after excluding the 200-review development subset; never overwritten by AI |
| Extension | 1,304 reviews = 1,704 − 400 by analysis_id |
| AI role | DeepSeek-V4-Pro PRE-CODING only |
| Verifier (internal label Human C) | 100% human verification of all 1,304 extension reviews; 28 failed pre-codes coded directly from review text |
| Auditor (internal label Human D) | Prespecified independent blind audit of 261 reviews (about 20%) |
| Audit sampling | Stratified by book × pre-coding any-substantive-code status; seed 20260829 |
| Blinding | Auditor did not see model predictions or verifier labels |
| Disagreements | Third-researcher adjudication |
| Failure handling | 28 failed DeepSeek outputs were not imputed; they were coded directly during full human verification |
| Auxiliary sampling-status assignment | A human researcher later reconstructed the auxiliary AI any-positive status for the 28 DeepSeek failures solely so that the prespecified audit-sampling rule could be applied; no verifier labels or final analytic labels were changed. |

***Note.*** *The protocol was frozen before the 1,304-review AI-assisted pre-coding run and was not modified in response to downstream results.*

**Supplementary Table S6. Independent verifier–auditor audit of the 1,304-review extension**

| **Index** | **Value** |
| --- | --- |
| Independent blind audit reviews | 261 |
| Binary code decisions compared | 10,179 |
| Agreed binary decisions | 9,645 |
| Overall code-cell agreement | 94.75% |
| Overall micro Cohen’s κ | 0.702 |
| Exact 39-code review matches | 59/261 |
| Reviews with ≥1 code disagreement | 202 |
| Disagreement code-cells sent to third-researcher adjudication | 534 |
| Deterministic C0 logic corrections after substantive-code adjudication | 9 audited records |

Note. A different researcher independently coded the prespecified 261-review blind audit sample while blinded to model and verifier labels. Human C and Human D are internal supplementary-file labels for the verifier and auditor, not additional researchers. All code-cell disagreements were isolated for third-researcher adjudication. For the 28 DeepSeek failures, the later Human researcher reconstruction affected only the auxiliary audit-sampling field and did not supply or alter final labels.

**Supplementary Table S7. Final 1,704 × 39 analytic matrix provenance and QC**

| **Provenance / QC item** | **N / status** |
| --- | --- |
| Locked Human Reference | 400 |
| Human-verified extension | 1,304 |
| Independent blind audit | 261 |
| Final review rows | 1,704 |
| Unique analysis_id values | 1,704 |
| 400 + 1,304 overlap | 0 (PASS) |
| Final C0 exclusivity logic | PASS |
| Original analytic-row order | Preserved |
| Audited records requiring deterministic C0 correction | 9 |

Note. The final 1,704-review matrix is the sole downstream source for final descriptive, comparative, and co-occurrence statistics. In S2 Data, final-label provenance is exposed through the exact variables final_label_provenance and independent_audit_applied.

# 5. Final statistical analysis and complete results

Final statistical analysis used the human-governed 1,704 × 39 matrix (*We Do Not Part*: n = 837; *Human Acts*: n = 867). Percentile bootstrap 95% confidence intervals used B = 10,000 review-level resamples (seed 20260836). Between-book tests used Fisher’s exact test when any expected 2 × 2 cell was <5 and Pearson chi-square otherwise. Benjamini–Hochberg FDR correction was applied as one family across 39 code comparisons and separately across 8 predefined code-group comparisons.

**Supplementary Table S8. Final statistical-analysis specification**

| **Item** | **Final specification** |
| --- | --- |
| Analysis unit | Review |
| Total N | 1704 |
| *We Do Not Part* | 837 |
| *Human Acts* | 867 |
| Final code variables | 39 binary codes (C0, C1.1–C8.5) |
| Predefined code-group variables | C1–C8; group = 1 if any member code = 1 |
| Bootstrap | 10,000 percentile resamples; seed 20260836 |
| Code multiple testing | Benjamini–Hochberg FDR across 39 code comparisons |
| Code-group multiple testing | Benjamini–Hochberg FDR across 8 predefined code-group comparisons |
| Difference direction | *We Do Not Part* − *Human Acts* |
| Co-occurrence | 38 substantive codes only; C0 excluded; phi/Jaccard/lift available |

**Supplementary Table S9. Complete prevalence and between-book comparisons for the 39 final reception codes**

| **Code** | **Code name** | **Overall** | ***We Do Not Part*** | ***Human Acts*** | **Difference (WDNP − HA)** | **p** | **q (BH-FDR)** | **Odds ratio** | **Phi / Cramer's V** |
| --- | --- | --- | --- | --- | --- | --- | --- | --- | --- |
| C0 | No codable reception content | 167 (9.8%; 95% CI 8.4%–11.3%) | 68 (8.1%; 95% CI 6.3%–9.9%) | 99 (11.4%; 95% CI 9.3%–13.6%) | -3.3 pp (95% CI -6.1 to -0.5) | 0.022 | 0.036 | 0.69 | -0.06 |
| C1.1 | Sadness / pain / grief | 241 (14.1%; 95% CI 12.5%–15.8%) | 78 (9.3%; 95% CI 7.4%–11.4%) | 163 (18.8%; 95% CI 16.3%–21.5%) | -9.5 pp (95% CI -12.8 to -6.2) | <0.001 | <0.001 | 0.44 | -0.14 |
| C1.2 | Heaviness / oppression / despair | 194 (11.4%; 95% CI 9.9%–12.9%) | 82 (9.8%; 95% CI 7.9%–11.9%) | 112 (12.9%; 95% CI 10.7%–15.2%) | -3.1 pp (95% CI -6.2 to -0.1) | 0.043 | 0.064 | 0.73 | -0.05 |
| C1.3 | Other salient / complex emotions | 158 (9.3%; 95% CI 7.9%–10.7%) | 43 (5.1%; 95% CI 3.7%–6.7%) | 115 (13.3%; 95% CI 11.1%–15.6%) | -8.1 pp (95% CI -10.8 to -5.4) | <0.001 | <0.001 | 0.35 | -0.14 |
| C1.4 | Embodied reception | 172 (10.1%; 95% CI 8.7%–11.6%) | 81 (9.7%; 95% CI 7.8%–11.7%) | 91 (10.5%; 95% CI 8.4%–12.6%) | -0.8 pp (95% CI -3.6 to +2.1) | 0.575 | 0.590 | 0.91 | -0.01 |
| C1.5 | Affective resonance / empathy / psychological proximity | 99 (5.8%; 95% CI 4.8%–6.9%) | 35 (4.2%; 95% CI 2.9%–5.6%) | 64 (7.4%; 95% CI 5.8%–9.1%) | -3.2 pp (95% CI -5.5 to -1.1) | 0.005 | 0.010 | 0.55 | -0.07 |
| C1.6 | Reported immersion / sense of presence | 88 (5.2%; 95% CI 4.1%–6.2%) | 31 (3.7%; 95% CI 2.5%–5.0%) | 57 (6.6%; 95% CI 5.0%–8.2%) | -2.9 pp (95% CI -5.0 to -0.8) | 0.007 | 0.014 | 0.55 | -0.06 |
| C1.7 | Affective distance / difficulty empathizing or identifying | 48 (2.8%; 95% CI 2.1%–3.6%) | 26 (3.1%; 95% CI 2.0%–4.3%) | 22 (2.5%; 95% CI 1.5%–3.6%) | +0.6 pp (95% CI -1.0 to +2.2) | 0.478 | 0.515 | 1.23 | 0.02 |
| C2.1 | Language / style appreciation or perception | 292 (17.1%; 95% CI 15.4%–19.0%) | 161 (19.2%; 95% CI 16.6%–22.0%) | 131 (15.1%; 95% CI 12.8%–17.5%) | +4.1 pp (95% CI +0.6 to +7.7) | 0.024 | 0.037 | 1.34 | 0.05 |
| C2.2 | Narrative / formal appreciation or perception | 260 (15.3%; 95% CI 13.6%–17.0%) | 109 (13.0%; 95% CI 10.8%–15.3%) | 151 (17.4%; 95% CI 14.9%–20.0%) | -4.4 pp (95% CI -7.8 to -1.0) | 0.012 | 0.021 | 0.71 | -0.06 |
| C2.3 | Imagery, title, symbolic meaning and visualization | 196 (11.5%; 95% CI 10.0%–13.0%) | 122 (14.6%; 95% CI 12.2%–17.1%) | 74 (8.5%; 95% CI 6.7%–10.4%) | +6.0 pp (95% CI +3.0 to +9.1) | <0.001 | <0.001 | 1.83 | 0.09 |
| C2.4 | Appraisal of trauma / violence representation | 230 (13.5%; 95% CI 11.9%–15.1%) | 78 (9.3%; 95% CI 7.4%–11.4%) | 152 (17.5%; 95% CI 15.1%–20.2%) | -8.2 pp (95% CI -11.4 to -4.9) | <0.001 | <0.001 | 0.48 | -0.12 |
| C2.5 | Negative formal / literary evaluation | 289 (17.0%; 95% CI 15.2%–18.8%) | 165 (19.7%; 95% CI 17.1%–22.5%) | 124 (14.3%; 95% CI 12.1%–16.7%) | +5.4 pp (95% CI +1.9 to +8.9) | 0.003 | 0.006 | 1.47 | 0.07 |
| C2.6 | Form-over-content / historical adequacy criticism | 110 (6.5%; 95% CI 5.3%–7.6%) | 60 (7.2%; 95% CI 5.5%–9.0%) | 50 (5.8%; 95% CI 4.3%–7.4%) | +1.4 pp (95% CI -0.9 to +3.7) | 0.239 | 0.292 | 1.26 | 0.03 |
| C2.7 | Genre / medium suitability judgment | 25 (1.5%; 95% CI 0.9%–2.1%) | 14 (1.7%; 95% CI 0.8%–2.6%) | 11 (1.3%; 95% CI 0.6%–2.1%) | +0.4 pp (95% CI -0.8 to +1.6) | 0.488 | 0.515 | 1.32 | 0.02 |
| C3.1 | General reading / comprehension difficulty | 77 (4.5%; 95% CI 3.6%–5.5%) | 68 (8.1%; 95% CI 6.3%–10.0%) | 9 (1.0%; 95% CI 0.5%–1.7%) | +7.1 pp (95% CI +5.1 to +9.0) | <0.001 | <0.001 | 8.43 | 0.17 |
| C3.2 | Fragmentation / abstraction / jumpiness difficulty | 79 (4.6%; 95% CI 3.6%–5.6%) | 72 (8.6%; 95% CI 6.8%–10.5%) | 7 (0.8%; 95% CI 0.2%–1.5%) | +7.8 pp (95% CI +5.8 to +9.8) | <0.001 | <0.001 | 11.56 | 0.19 |
| C3.3 | Person / viewpoint / subject-identification difficulty | 35 (2.1%; 95% CI 1.4%–2.8%) | 20 (2.4%; 95% CI 1.4%–3.5%) | 15 (1.7%; 95% CI 0.9%–2.7%) | +0.7 pp (95% CI -0.6 to +2.0) | 0.337 | 0.376 | 1.39 | 0.02 |
| C3.4 | Temporal / scene / reality-imagination transition difficulty | 19 (1.1%; 95% CI 0.6%–1.6%) | 17 (2.0%; 95% CI 1.1%–3.0%) | 2 (0.2%; 95% CI 0.0%–0.6%) | +1.8 pp (95% CI +0.8 to +2.9) | <0.001 | <0.001 | 8.97 | 0.09 |
| C4.1 | Historical knowledge barrier | 32 (1.9%; 95% CI 1.3%–2.5%) | 21 (2.5%; 95% CI 1.6%–3.6%) | 11 (1.3%; 95% CI 0.6%–2.1%) | +1.2 pp (95% CI -0.1 to +2.5) | 0.059 | 0.086 | 2.00 | 0.05 |
| C4.2 | Active historical contextualization | 23 (1.3%; 95% CI 0.8%–1.9%) | 8 (1.0%; 95% CI 0.4%–1.7%) | 15 (1.7%; 95% CI 0.9%–2.7%) | -0.8 pp (95% CI -1.8 to +0.3) | 0.166 | 0.223 | 0.55 | -0.03 |
| C4.3 | New historical awareness / educational value | 69 (4.0%; 95% CI 3.2%–5.0%) | 41 (4.9%; 95% CI 3.5%–6.5%) | 28 (3.2%; 95% CI 2.1%–4.5%) | +1.7 pp (95% CI -0.2 to +3.5) | 0.081 | 0.112 | 1.54 | 0.04 |
| C5.1 | Translation evaluation | 175 (10.3%; 95% CI 8.9%–11.8%) | 153 (18.3%; 95% CI 15.8%–20.9%) | 22 (2.5%; 95% CI 1.5%–3.7%) | +15.7 pp (95% CI +12.9 to +18.6) | <0.001 | <0.001 | 8.59 | 0.26 |
| C5.2 | Translation-induced difficulty | 60 (3.5%; 95% CI 2.7%–4.5%) | 58 (6.9%; 95% CI 5.3%–8.7%) | 2 (0.2%; 95% CI 0.0%–0.6%) | +6.7 pp (95% CI +5.0 to +8.5) | <0.001 | <0.001 | 32.20 | 0.18 |
| C5.3 | Paratext / edition design evaluation | 15 (0.9%; 95% CI 0.5%–1.3%) | 10 (1.2%; 95% CI 0.5%–2.0%) | 5 (0.6%; 95% CI 0.1%–1.2%) | +0.6 pp (95% CI -0.2 to +1.6) | 0.172 | 0.224 | 2.08 | 0.03 |
| C6.1 | Understanding of long-term / transgenerational trauma | 232 (13.6%; 95% CI 12.0%–15.3%) | 97 (11.6%; 95% CI 9.4%–13.7%) | 135 (15.6%; 95% CI 13.1%–18.0%) | -4.0 pp (95% CI -7.1 to -0.7) | 0.017 | 0.028 | 0.71 | -0.06 |
| C6.2 | Memory / non-forgetting / retelling ethics | 264 (15.5%; 95% CI 13.8%–17.2%) | 120 (14.3%; 95% CI 11.9%–16.7%) | 144 (16.6%; 95% CI 14.2%–19.1%) | -2.3 pp (95% CI -5.6 to +1.2) | 0.195 | 0.245 | 0.84 | -0.03 |
| C6.3 | State violence / responsibility / justice reflection | 209 (12.3%; 95% CI 10.7%–13.8%) | 44 (5.3%; 95% CI 3.8%–6.8%) | 165 (19.0%; 95% CI 16.4%–21.7%) | -13.8 pp (95% CI -16.8 to -10.8) | <0.001 | <0.001 | 0.24 | -0.21 |
| C6.4 | Human nature / violence / dignity / existential reflection | 251 (14.7%; 95% CI 13.1%–16.4%) | 54 (6.5%; 95% CI 4.8%–8.1%) | 197 (22.7%; 95% CI 20.0%–25.5%) | -16.3 pp (95% CI -19.5 to -13.0) | <0.001 | <0.001 | 0.23 | -0.23 |
| C6.5 | Literature as memory / witness / historical writing | 195 (11.4%; 95% CI 9.9%–13.0%) | 61 (7.3%; 95% CI 5.6%–9.1%) | 134 (15.5%; 95% CI 13.1%–17.9%) | -8.2 pp (95% CI -11.1 to -5.2) | <0.001 | <0.001 | 0.43 | -0.13 |
| C6.6 | Reader attribution/evaluation of author intent, position, or persona | 248 (14.6%; 95% CI 12.9%–16.3%) | 103 (12.3%; 95% CI 10.0%–14.6%) | 145 (16.7%; 95% CI 14.3%–19.3%) | -4.4 pp (95% CI -7.7 to -1.1) | 0.010 | 0.018 | 0.70 | -0.06 |
| C7.1 | Comparison with Han Kang's other works | 237 (13.9%; 95% CI 12.3%–15.6%) | 152 (18.2%; 95% CI 15.5%–20.8%) | 85 (9.8%; 95% CI 7.8%–11.9%) | +8.4 pp (95% CI +5.1 to +11.7) | <0.001 | <0.001 | 2.04 | 0.12 |
| C7.2 | Intertextual comparison with other works / media | 170 (10.0%; 95% CI 8.6%–11.4%) | 54 (6.5%; 95% CI 4.9%–8.1%) | 116 (13.4%; 95% CI 11.1%–15.7%) | -6.9 pp (95% CI -9.7 to -4.1) | <0.001 | <0.001 | 0.45 | -0.12 |
| C7.3 | Cross-cultural / cross-historical association and world-literary positioning | 105 (6.2%; 95% CI 5.0%–7.3%) | 31 (3.7%; 95% CI 2.5%–5.0%) | 74 (8.5%; 95% CI 6.7%–10.4%) | -4.8 pp (95% CI -7.2 to -2.6) | <0.001 | <0.001 | 0.41 | -0.10 |
| C8.1 | Overall evaluation / value judgment | 381 (22.4%; 95% CI 20.4%–24.4%) | 186 (22.2%; 95% CI 19.5%–25.0%) | 195 (22.5%; 95% CI 19.8%–25.3%) | -0.3 pp (95% CI -4.2 to +3.6) | 0.894 | 0.894 | 0.98 | -0.00 |
| C8.2 | Reading motivation / expectation | 88 (5.2%; 95% CI 4.1%–6.2%) | 38 (4.5%; 95% CI 3.2%–6.0%) | 50 (5.8%; 95% CI 4.3%–7.4%) | -1.2 pp (95% CI -3.4 to +0.9) | 0.253 | 0.298 | 0.78 | -0.03 |
| C8.3 | Reading attraction, boredom, fatigue, and retention | 148 (8.7%; 95% CI 7.4%–10.0%) | 105 (12.5%; 95% CI 10.3%–14.8%) | 43 (5.0%; 95% CI 3.6%–6.5%) | +7.6 pp (95% CI +5.0 to +10.2) | <0.001 | <0.001 | 2.75 | 0.13 |
| C8.4 | Reading behavior / coping strategy | 204 (12.0%; 95% CI 10.4%–13.6%) | 93 (11.1%; 95% CI 9.1%–13.4%) | 111 (12.8%; 95% CI 10.6%–15.1%) | -1.7 pp (95% CI -4.8 to +1.4) | 0.282 | 0.324 | 0.85 | -0.03 |
| C8.5 | Meta-reception / evaluation frames | 44 (2.6%; 95% CI 1.9%–3.3%) | 11 (1.3%; 95% CI 0.6%–2.2%) | 33 (3.8%; 95% CI 2.5%–5.1%) | -2.5 pp (95% CI -4.0 to -1.0) | 0.001 | 0.003 | 0.34 | -0.08 |

Note. Overall and within-work cells show n (prevalence; bootstrap 95% CI). Difference is *We Do Not Part* minus *Human Acts* in percentage points. q values use Benjamini–Hochberg FDR correction across the 39 code comparisons.

**Supplementary Table S10. Predefined code-group prevalence and between-book comparisons**

| **Code group** | **Code-group name** | **Member codes** | **Overall** | ***We Do Not Part*** | ***Human Acts*** | **Difference (WDNP − HA)** | **p** | **q (BH-FDR)** | **Odds ratio** | **Phi / Cramer's V** |
| --- | --- | --- | --- | --- | --- | --- | --- | --- | --- | --- |
| C1 | Affective and embodied responses | C1.1;C1.2;C1.3;C1.4;C1.5;C1.6;C1.7 | 625 (36.7%; 95% CI 34.4%–39.0%) | 261 (31.2%; 95% CI 28.1%–34.4%) | 364 (42.0%; 95% CI 38.8%–45.2%) | -10.8 pp (95% CI -15.5 to -6.3) | <0.001 | <0.001 | 0.63 | -0.11 |
| C2 | Literary form and aesthetic perception | C2.1;C2.2;C2.3;C2.4;C2.5;C2.6;C2.7 | 836 (49.1%; 95% CI 46.7%–51.3%) | 435 (52.0%; 95% CI 48.5%–55.3%) | 401 (46.3%; 95% CI 42.8%–49.5%) | +5.7 pp (95% CI +0.9 to +10.5) | 0.018 | 0.024 | 1.26 | 0.06 |
| C3 | Reading and comprehension difficulties | C3.1;C3.2;C3.3;C3.4 | 187 (11.0%; 95% CI 9.5%–12.4%) | 156 (18.6%; 95% CI 16.1%–21.3%) | 31 (3.6%; 95% CI 2.4%–4.8%) | +15.1 pp (95% CI +12.1 to +17.9) | <0.001 | <0.001 | 6.18 | 0.24 |
| C4 | Knowledge, history, and cognitive gains | C4.1;C4.2;C4.3 | 103 (6.0%; 95% CI 4.9%–7.2%) | 63 (7.5%; 95% CI 5.9%–9.3%) | 40 (4.6%; 95% CI 3.2%–6.1%) | +2.9 pp (95% CI +0.7 to +5.2) | 0.012 | 0.019 | 1.68 | 0.06 |
| C5 | Translation and edition reception | C5.1;C5.2;C5.3 | 189 (11.1%; 95% CI 9.6%–12.6%) | 162 (19.4%; 95% CI 16.6%–22.1%) | 27 (3.1%; 95% CI 2.0%–4.3%) | +16.2 pp (95% CI +13.3 to +19.1) | <0.001 | <0.001 | 7.47 | 0.26 |
| C6 | Ethical, mnemonic, political, and humanistic reflection | C6.1;C6.2;C6.3;C6.4;C6.5;C6.6 | 706 (41.4%; 95% CI 39.1%–43.7%) | 253 (30.2%; 95% CI 27.1%–33.3%) | 453 (52.2%; 95% CI 48.9%–55.6%) | -22.0 pp (95% CI -26.7 to -17.5) | <0.001 | <0.001 | 0.40 | -0.22 |
| C7 | Intertextual, intermedial, and cross-cultural associations | C7.1;C7.2;C7.3 | 427 (25.1%; 95% CI 22.9%–27.1%) | 205 (24.5%; 95% CI 21.6%–27.5%) | 222 (25.6%; 95% CI 22.7%–28.6%) | -1.1 pp (95% CI -5.1 to +3.0) | 0.596 | 0.681 | 0.94 | -0.01 |
| C8 | Overall evaluation and reading behavior | C8.1;C8.2;C8.3;C8.4;C8.5 | 649 (38.1%; 95% CI 35.9%–40.4%) | 321 (38.4%; 95% CI 35.0%–41.6%) | 328 (37.8%; 95% CI 34.7%–41.2%) | +0.5 pp (95% CI -4.1 to +5.2) | 0.825 | 0.825 | 1.02 | 0.01 |

Note. A predefined code group was present when at least one member code was positive. Difference is *We Do Not Part* minus *Human Acts* in percentage points. q values use Benjamini–Hochberg FDR correction across the eight predefined code-group comparisons.

# 6. Supplementary figures

Supplementary Figure S2 is based on the final 1,704-review matrix and summarizes pairwise co-occurrence among the 38 substantive reception codes.

**Supplementary Figure S2.** Co-occurrence structure of substantive reception codes across the full corpus.

Cells show pairwise phi coefficients among the 38 substantive reception codes across all 1,704 reviews. C0 is excluded because it denotes the absence of substantive reception content. The diagonal is omitted because self-correlation is structurally 1.0.

# 7. Sensitivity analyses

Two secondary sensitivity analyses were conducted using the same review-level test-selection rule as the primary analysis. First, all 39 between-book code comparisons were repeated within the fully human-coded and adjudicated 400-review Human Reference (200 reviews per book). Second, the 167 C0 reviews were removed and the 38 substantive-code comparisons were repeated in the remaining 1,537 reviews. Benjamini–Hochberg correction was applied within each sensitivity-analysis family.

**Supplementary Table S11. Fully human-coded 400-review sensitivity analysis**

| **Code** | **Code name** | ***We Do Not Part* (n=200)** | ***Human Acts* (n=200)** | **Difference (pp)** | **Test** | **p** | **q (BH-FDR)** | **FDR < .05** |
| --- | --- | --- | --- | --- | --- | --- | --- | --- |
| C0 | No codable reception content | 9 (4.5%) | 24 (12.0%) | -7.5 | Pearson chi-square | 0.006 | 0.030 | Yes |
| C1.1 | Sadness / pain / grief | 29 (14.5%) | 47 (23.5%) | -9.0 | Pearson chi-square | 0.022 | 0.082 | No |
| C1.2 | Heaviness / oppression / despair | 19 (9.5%) | 20 (10.0%) | -0.5 | Pearson chi-square | 0.866 | 0.913 | No |
| C1.3 | Other salient / complex emotions | 21 (10.5%) | 37 (18.5%) | -8.0 | Pearson chi-square | 0.023 | 0.082 | No |
| C1.4 | Embodied reception | 24 (12.0%) | 19 (9.5%) | +2.5 | Pearson chi-square | 0.420 | 0.584 | No |
| C1.5 | Affective resonance / empathy / psychological proximity | 14 (7.0%) | 20 (10.0%) | -3.0 | Pearson chi-square | 0.282 | 0.500 | No |
| C1.6 | Reported immersion / sense of presence | 7 (3.5%) | 11 (5.5%) | -2.0 | Pearson chi-square | 0.335 | 0.522 | No |
| C1.7 | Affective distance / difficulty empathizing or identifying | 6 (3.0%) | 8 (4.0%) | -1.0 | Pearson chi-square | 0.586 | 0.693 | No |
| C2.1 | Language / style appreciation or perception | 48 (24.0%) | 36 (18.0%) | +6.0 | Pearson chi-square | 0.141 | 0.289 | No |
| C2.2 | Narrative / formal appreciation or perception | 38 (19.0%) | 47 (23.5%) | -4.5 | Pearson chi-square | 0.271 | 0.500 | No |
| C2.3 | Imagery, title, symbolic meaning and visualization | 30 (15.0%) | 33 (16.5%) | -1.5 | Pearson chi-square | 0.681 | 0.758 | No |
| C2.4 | Appraisal of trauma / violence representation | 36 (18.0%) | 59 (29.5%) | -11.5 | Pearson chi-square | 0.007 | 0.030 | Yes |
| C2.5 | Negative formal / literary evaluation | 46 (23.0%) | 36 (18.0%) | +5.0 | Pearson chi-square | 0.216 | 0.420 | No |
| C2.6 | Form-over-content / historical adequacy criticism | 15 (7.5%) | 12 (6.0%) | +1.5 | Pearson chi-square | 0.550 | 0.670 | No |
| C2.7 | Genre / medium suitability judgment | 1 (0.5%) | 3 (1.5%) | -1.0 | Fisher exact | 0.623 | 0.715 | No |
| C3.1 | General reading / comprehension difficulty | 24 (12.0%) | 2 (1.0%) | +11.0 | Pearson chi-square | <0.001 | <0.001 | Yes |
| C3.2 | Fragmentation / abstraction / jumpiness difficulty | 13 (6.5%) | 2 (1.0%) | +5.5 | Pearson chi-square | 0.004 | 0.025 | Yes |
| C3.3 | Person / viewpoint / subject-identification difficulty | 6 (3.0%) | 4 (2.0%) | +1.0 | Pearson chi-square | 0.522 | 0.657 | No |
| C3.4 | Temporal / scene / reality-imagination transition difficulty | 6 (3.0%) | 0 (0.0%) | +3.0 | Fisher exact | 0.030 | 0.098 | No |
| C4.1 | Historical knowledge barrier | 5 (2.5%) | 2 (1.0%) | +1.5 | Fisher exact | 0.449 | 0.604 | No |
| C4.2 | Active historical contextualization | 4 (2.0%) | 1 (0.5%) | +1.5 | Fisher exact | 0.372 | 0.537 | No |
| C4.3 | New historical awareness / educational value | 7 (3.5%) | 4 (2.0%) | +1.5 | Pearson chi-square | 0.359 | 0.537 | No |
| C5.1 | Translation evaluation | 41 (20.5%) | 6 (3.0%) | +17.5 | Pearson chi-square | <0.001 | <0.001 | Yes |
| C5.2 | Translation-induced difficulty | 11 (5.5%) | 1 (0.5%) | +5.0 | Pearson chi-square | 0.003 | 0.025 | Yes |
| C5.3 | Paratext / edition design evaluation | 4 (2.0%) | 0 (0.0%) | +2.0 | Fisher exact | 0.123 | 0.289 | No |
| C6.1 | Understanding of long-term / transgenerational trauma | 27 (13.5%) | 32 (16.0%) | -2.5 | Pearson chi-square | 0.481 | 0.625 | No |
| C6.2 | Memory / non-forgetting / retelling ethics | 31 (15.5%) | 31 (15.5%) | +0.0 | Pearson chi-square | 1.000 | 1.000 | No |
| C6.3 | State violence / responsibility / justice reflection | 16 (8.0%) | 50 (25.0%) | -17.0 | Pearson chi-square | <0.001 | <0.001 | Yes |
| C6.4 | Human nature / violence / dignity / existential reflection | 13 (6.5%) | 52 (26.0%) | -19.5 | Pearson chi-square | <0.001 | <0.001 | Yes |
| C6.5 | Literature as memory / witness / historical writing | 17 (8.5%) | 30 (15.0%) | -6.5 | Pearson chi-square | 0.044 | 0.127 | No |
| C6.6 | Reader attribution/evaluation of author intent, position, or persona | 33 (16.5%) | 41 (20.5%) | -4.0 | Pearson chi-square | 0.303 | 0.503 | No |
| C7.1 | Comparison with Han Kang's other works | 37 (18.5%) | 18 (9.0%) | +9.5 | Pearson chi-square | 0.006 | 0.030 | Yes |
| C7.2 | Intertextual comparison with other works / media | 21 (10.5%) | 31 (15.5%) | -5.0 | Pearson chi-square | 0.137 | 0.289 | No |
| C7.3 | Cross-cultural / cross-historical association and world-literary positioning | 12 (6.0%) | 20 (10.0%) | -4.0 | Pearson chi-square | 0.140 | 0.289 | No |
| C8.1 | Overall evaluation / value judgment | 58 (29.0%) | 49 (24.5%) | +4.5 | Pearson chi-square | 0.309 | 0.503 | No |
| C8.2 | Reading motivation / expectation | 11 (5.5%) | 11 (5.5%) | +0.0 | Pearson chi-square | 1.000 | 1.000 | No |
| C8.3 | Reading attraction, boredom, fatigue, and retention | 26 (13.0%) | 14 (7.0%) | +6.0 | Pearson chi-square | 0.046 | 0.127 | No |
| C8.4 | Reading behavior / coping strategy | 25 (12.5%) | 23 (11.5%) | +1.0 | Pearson chi-square | 0.758 | 0.821 | No |
| C8.5 | Meta-reception / evaluation frames | 7 (3.5%) | 14 (7.0%) | -3.5 | Pearson chi-square | 0.117 | 0.289 | No |

Note. Nine of the 39 codes remained significant after FDR correction. The strongest contrasts included translation evaluation (C5.1, +17.5 pp), human-nature/violence/dignity/existential reflection (C6.4, −19.5 pp), state-violence/responsibility/justice reflection (C6.3, −17.0 pp), and general reading/comprehension difficulty (C3.1, +11.0 pp), all in the same direction as the full-corpus estimates.

**Supplementary Table S12. C0-excluded sensitivity analysis of the 38 substantive codes**

| **Code** | **Code name** | ***We Do Not Part* (n=769)** | ***Human Acts* (n=768)** | **Difference (pp)** | **Test** | **p** | **q (BH-FDR)** | **FDR < .05** |
| --- | --- | --- | --- | --- | --- | --- | --- | --- |
| C1.1 | Sadness / pain / grief | 78 (10.1%) | 163 (21.2%) | -11.1 | Pearson chi-square | <0.001 | <0.001 | Yes |
| C1.2 | Heaviness / oppression / despair | 82 (10.7%) | 112 (14.6%) | -3.9 | Pearson chi-square | 0.021 | 0.033 | Yes |
| C1.3 | Other salient / complex emotions | 43 (5.6%) | 115 (15.0%) | -9.4 | Pearson chi-square | <0.001 | <0.001 | Yes |
| C1.4 | Embodied reception | 81 (10.5%) | 91 (11.8%) | -1.3 | Pearson chi-square | 0.413 | 0.449 | No |
| C1.5 | Affective resonance / empathy / psychological proximity | 35 (4.6%) | 64 (8.3%) | -3.8 | Pearson chi-square | 0.003 | 0.005 | Yes |
| C1.6 | Reported immersion / sense of presence | 31 (4.0%) | 57 (7.4%) | -3.4 | Pearson chi-square | 0.004 | 0.008 | Yes |
| C1.7 | Affective distance / difficulty empathizing or identifying | 26 (3.4%) | 22 (2.9%) | +0.5 | Pearson chi-square | 0.561 | 0.576 | No |
| C2.1 | Language / style appreciation or perception | 161 (20.9%) | 131 (17.1%) | +3.9 | Pearson chi-square | 0.053 | 0.080 | No |
| C2.2 | Narrative / formal appreciation or perception | 109 (14.2%) | 151 (19.7%) | -5.5 | Pearson chi-square | 0.004 | 0.008 | Yes |
| C2.3 | Imagery, title, symbolic meaning and visualization | 122 (15.9%) | 74 (9.6%) | +6.2 | Pearson chi-square | <0.001 | <0.001 | Yes |
| C2.4 | Appraisal of trauma / violence representation | 78 (10.1%) | 152 (19.8%) | -9.6 | Pearson chi-square | <0.001 | <0.001 | Yes |
| C2.5 | Negative formal / literary evaluation | 165 (21.5%) | 124 (16.1%) | +5.3 | Pearson chi-square | 0.008 | 0.013 | Yes |
| C2.6 | Form-over-content / historical adequacy criticism | 60 (7.8%) | 50 (6.5%) | +1.3 | Pearson chi-square | 0.326 | 0.375 | No |
| C2.7 | Genre / medium suitability judgment | 14 (1.8%) | 11 (1.4%) | +0.4 | Pearson chi-square | 0.547 | 0.576 | No |
| C3.1 | General reading / comprehension difficulty | 68 (8.8%) | 9 (1.2%) | +7.7 | Pearson chi-square | <0.001 | <0.001 | Yes |
| C3.2 | Fragmentation / abstraction / jumpiness difficulty | 72 (9.4%) | 7 (0.9%) | +8.5 | Pearson chi-square | <0.001 | <0.001 | Yes |
| C3.3 | Person / viewpoint / subject-identification difficulty | 20 (2.6%) | 15 (2.0%) | +0.6 | Pearson chi-square | 0.395 | 0.441 | No |
| C3.4 | Temporal / scene / reality-imagination transition difficulty | 17 (2.2%) | 2 (0.3%) | +2.0 | Pearson chi-square | <0.001 | 0.001 | Yes |
| C4.1 | Historical knowledge barrier | 21 (2.7%) | 11 (1.4%) | +1.3 | Pearson chi-square | 0.075 | 0.109 | No |
| C4.2 | Active historical contextualization | 8 (1.0%) | 15 (2.0%) | -0.9 | Pearson chi-square | 0.141 | 0.184 | No |
| C4.3 | New historical awareness / educational value | 41 (5.3%) | 28 (3.6%) | +1.7 | Pearson chi-square | 0.111 | 0.150 | No |
| C5.1 | Translation evaluation | 153 (19.9%) | 22 (2.9%) | +17.0 | Pearson chi-square | <0.001 | <0.001 | Yes |
| C5.2 | Translation-induced difficulty | 58 (7.5%) | 2 (0.3%) | +7.3 | Pearson chi-square | <0.001 | <0.001 | Yes |
| C5.3 | Paratext / edition design evaluation | 10 (1.3%) | 5 (0.7%) | +0.6 | Pearson chi-square | 0.195 | 0.232 | No |
| C6.1 | Understanding of long-term / transgenerational trauma | 97 (12.6%) | 135 (17.6%) | -5.0 | Pearson chi-square | 0.007 | 0.011 | Yes |
| C6.2 | Memory / non-forgetting / retelling ethics | 120 (15.6%) | 144 (18.8%) | -3.1 | Pearson chi-square | 0.102 | 0.144 | No |
| C6.3 | State violence / responsibility / justice reflection | 44 (5.7%) | 165 (21.5%) | -15.8 | Pearson chi-square | <0.001 | <0.001 | Yes |
| C6.4 | Human nature / violence / dignity / existential reflection | 54 (7.0%) | 197 (25.7%) | -18.6 | Pearson chi-square | <0.001 | <0.001 | Yes |
| C6.5 | Literature as memory / witness / historical writing | 61 (7.9%) | 134 (17.4%) | -9.5 | Pearson chi-square | <0.001 | <0.001 | Yes |
| C6.6 | Reader attribution/evaluation of author intent, position, or persona | 103 (13.4%) | 145 (18.9%) | -5.5 | Pearson chi-square | 0.003 | 0.007 | Yes |
| C7.1 | Comparison with Han Kang's other works | 152 (19.8%) | 85 (11.1%) | +8.7 | Pearson chi-square | <0.001 | <0.001 | Yes |
| C7.2 | Intertextual comparison with other works / media | 54 (7.0%) | 116 (15.1%) | -8.1 | Pearson chi-square | <0.001 | <0.001 | Yes |
| C7.3 | Cross-cultural / cross-historical association and world-literary positioning | 31 (4.0%) | 74 (9.6%) | -5.6 | Pearson chi-square | <0.001 | <0.001 | Yes |
| C8.1 | Overall evaluation / value judgment | 186 (24.2%) | 195 (25.4%) | -1.2 | Pearson chi-square | 0.585 | 0.585 | No |
| C8.2 | Reading motivation / expectation | 38 (4.9%) | 50 (6.5%) | -1.6 | Pearson chi-square | 0.186 | 0.227 | No |
| C8.3 | Reading attraction, boredom, fatigue, and retention | 105 (13.7%) | 43 (5.6%) | +8.1 | Pearson chi-square | <0.001 | <0.001 | Yes |
| C8.4 | Reading behavior / coping strategy | 93 (12.1%) | 111 (14.5%) | -2.4 | Pearson chi-square | 0.173 | 0.219 | No |
| C8.5 | Meta-reception / evaluation frames | 11 (1.4%) | 33 (4.3%) | -2.9 | Pearson chi-square | <0.001 | 0.002 | Yes |

Note. Removing C0 left 1,537 reviews. Twenty-four of the 38 substantive codes met q < .05. Relative to the primary analysis, 23 of the 24 substantive codes that were significant remained significant; C2.1 moved above the FDR threshold (q = .080), while C1.2 moved below it (q = .033). Effect directions did not change.

# 8. Reproducibility package, code, and data access

The supporting materials document the current codebook-development, validation, production, audit, and final-analysis chain. This S1 File contains the public-facing operational representation of the frozen 39-code codebook, two development-stage codebook prompts, the construction and adjudication of the 400-review Human Reference, human reliability evidence, blind DeepSeek benchmark summaries, the frozen 1,304-review production protocol, verifier-auditor audit evidence, final-matrix provenance, complete statistical tables, sensitivity analyses, and Supplementary Fig S2.

S2 Data is the de-identified English-language analytic matrix used for the quantitative results. It contains analysis_id, book_title, all 39 final binary code variables, final_label_provenance, and independent_audit_applied. final_label_provenance distinguishes the 400 adjudicated Human Reference records from the 1,304 human-verified extension records, while independent_audit_applied identifies the 261 extension reviews included in the independent blind audit. Verbatim Douban review text, usernames, user IDs, profile URLs, and other direct user identifiers are not included or redistributed.

## 8.1. Accompanying S3 File reproducibility code archive

S3 File is the separate reproducibility code archive accompanying the submission. It contains README.md, requirements.txt, and modular Python scripts for public-data validation, 400-review human-coder reliability, two blind DeepSeek-V4-Pro benchmark reproductions, the 261-review independent audit, key-number checks, and reproduction of the final statistical analyses and figures.

The archive also contains de-identified, machine-readable auxiliary files needed to reproduce the validation and audit results: a public machine-readable representation of the frozen 39-code operational codebook (code IDs and names, definitions, inclusion criteria, exclusion criteria, and boundary rules; source-linked illustrative example fields omitted); the 400-review binary coding matrices from Human Coder A and Human Coder B; the adjudicated 400-review Human Reference; the two DeepSeek benchmark label matrices; the Human C verifier and Human D blind-auditor label matrices for the 261-review audit; and the adjudicated audit-final label matrix. These files contain analysis IDs and coding variables or status fields only; verbatim review text and direct user identifiers are not included.

Together with S2 Data, S3 File reproduces the locked-matrix quality checks; the reported human reliability, model-benchmark, and audit agreement summaries; the 39-code and eight predefined code-group prevalence estimates; percentile bootstrap 95% confidence intervals (10,000 review-level resamples; seed 20260836); between-book Fisher exact/Pearson chi-square tests and Benjamini-Hochberg corrections; pairwise phi/Jaccard/lift co-occurrence statistics; the two sensitivity analyses; Figures 1, 2, and 4; and Supplementary Fig S2. The public reproduction package does not reacquire Douban reviews or rerun external LLM API calls. Those upstream stages are documented through the production-prompt transcript with source-linked example wording redacted, the two development-stage prompt records with embedded review examples omitted where necessary, the public operational codebook, de-identified benchmark and audit label matrices, and the human-governed audit trail in S1 File.
